# Oncolytic virus OHSV2 induces pyroptosis in bladder cancer cells via the TLR4/NLRP3/Caspase-1/GSDMD pathway

**DOI:** 10.1186/s43556-026-00506-4

**Published:** 2026-07-28

**Authors:** Jinzhou Xu, Yifan Xiong, Chenqian Liu, Jianxuan Sun, Ye An, Zhiyu Xia, Binlei Liu, Jia Hu, Qidong Xia, Shaogang Wang

**Affiliations:** 1 Department and Institute of Urology, Tongji Hospital, Tongji Medical College, Huazhong University of Science and Technology, Wuhan, China; 2 Hubei Provincial Clinical Research Center for Minimally Invasive Treatment of Urology, Wuhan, China; 3 Laboratory of Signaling and Gene Regulation, Cecil H. and Ida Green Center for Reproductive Biology Sciences, University of Texas Southwestern Medical Center, Dallas, USA; 4 National “111” Center for Cellular Regulation and Molecular Pharmaceutics, Key Laboratory of Fermentation Engineering (Ministry of Education), Hubei Provincial Cooperative Innovation Center of Industrial Fermentation, Hubei Key Laboratory of Industrial Microbiology, Hubei University of Technology, Wuhan, China

**Keywords:** Oncolytic virus, Bladder cancer, Pyroptosis, TLR4, NLRP3

## Abstract

Bladder cancer remains a significant clinical challenge due to high recurrence and progression rates, necessitating novel therapeutic strategies. Oncolytic viruses, such as the herpes simplex virus type 2-based OHSV2, have demonstrated promising antitumor effects through direct oncolysis and immune activation. This study investigated the efficacy, safety, and molecular mechanisms of OHSV2 in the treatment of bladder cancer. In vitro and in vivo experiments demonstrated that OHSV2 potently inhibited bladder cancer cell proliferation, migration, and clonogenicity in a dose-dependent manner, while exhibiting a favorable safety profile. Critically, OHSV2 treatment triggered a pro-inflammatory tumor immune microenvironment, characterized by increased infiltration and activation of CD8^+^ T cells. Mechanistically, OHSV2 induced pyroptosis in bladder cancer cells via the canonical Caspase-1/Gasdermin D (GSDMD) pathway, accompanied by increased interleukin-18 (IL-18), interleukin-1β (IL-1β), and lactate dehydrogenase (LDH) release. Further analysis identified NOD-like receptor family pyrin domain containing 3 (NLRP3) as the key upstream pattern recognition receptor for Caspase-1/GSDMD activation, and Toll-like receptor 4 (TLR4) as a critical mediator of NLRP3-dependent pyroptosis. Inhibition of TLR4 or NLRP3 partially reversed OHSV2-induced cytotoxicity, confirming their functional roles. Additionally, combining OHSV2 with the TLR4 agonist enhanced pyroptosis in a subcutaneous xenograft model, amplified antitumor immunity, and improved tumor control in vivo, suggesting potential synergism for clinical translation. These findings elucidate a novel TLR4/NLRP3/Caspase-1/GSDMD axis as the core mechanism behind the antitumor effect of OHSV2 and propose a rationale for its combination with immunomodulators to improve outcomes in bladder cancer.

**Supplementary Information:**

The online version contains supplementary material available at 10.1186/s43556-026-00506-4.

## Introduction

Bladder cancer (BCa) represents the second most prevalent urological malignancy worldwide, with approximately 614,000 newly diagnosed cases and 200,000 associated deaths reported annually in 2022 [1]. Clinically, this malignancy is classified into two distinct subtypes based on invasion depth: non-muscle-invasive bladder cancer (NMIBC) and muscle-invasive bladder cancer (MIBC), accounting for approximately 70% to 75% and 20% to 25% of cases, respectively. For NMIBC, the 5-year recurrence rate ranges from 50% to 70%, and the risk of progression in high-risk NMIBC patients can be as high as 40% to 50% [2]. Moreover, the prognosis for patients with MIBC is significantly worse than that for patients with NMIBC. For those with regional-stage MIBC, the five-year relative survival rate is approximately 50% to 60%; however, once distant metastasis occurs, the five-year survival rate declines sharply to 6% to 15% [3]. Therefore, the development of novel therapeutic strategies for bladder cancer remains an urgent clinical imperative.

Oncolytic viruses (OVs) are viruses that occur naturally or have been genetically modified to selectively infect, replicate in, and destroy tumor cells while sparing normal cells [4]. OHSV2 represents a strategically engineered oncolytic virus with distinct features that position it as a compelling candidate for investigation. It is built on the herpes simplex virus type 2 (HSV-2) backbone, which offers a larger genomic capacity for transgene insertion compared to commonly used herpes simplex virus type 1 (HSV-1) vectors, facilitating its armament with therapeutic payloads. To optimize its therapeutic index, OHSV2 incorporates key safety modifications: deletion of the neurovirulence gene ICP34.5 reduces pathogenicity, while deletion of the immune evasion gene ICP47 enhances antigen presentation, potentially fostering a more robust anti-tumor immune response. Furthermore, OHSV2 is armed with human granulocyte–macrophage colony-stimulating factor (GM-CSF). This integrated design has garnered significant translational recognition, including Orphan Drug Designation (ODD) and Fast Track Designation (FTD) from the U.S. FDA. However, despite its promising profile and documented activity in other malignancies, the efficacy of OHSV2 in bladder cancer, a disease where local instillation therapy is standard, remains unexamined [5].

The antitumor effects of oncolytic viruses are attributed not only to direct lysis but also to the induction of immunogenic cell death, which activates systemic antitumor immunity [6]. Pyroptosis is a lytic process mediated by gasdermin family proteins and can lead to the release of pro-inflammatory cytokines and potent immune activation [7]. This mechanism has been implicated in the efficacy of some oncolytic viruses, but whether it contributes to OHSV2’s action in bladder cancer remains unknown [8].

Therefore, this study will confirm the efficacy and safety of OHSV2 in the treatment of bladder cancer and to investigate whether its antitumor effect involves the induction of pyroptosis. Furthermore, we sought to elucidate the underlying molecular mechanisms to provide a rationale for potential combination therapies.

## Results

### Verification of the tumor control effect of OHSV2 on bladder cancer

**Fig. 1:**
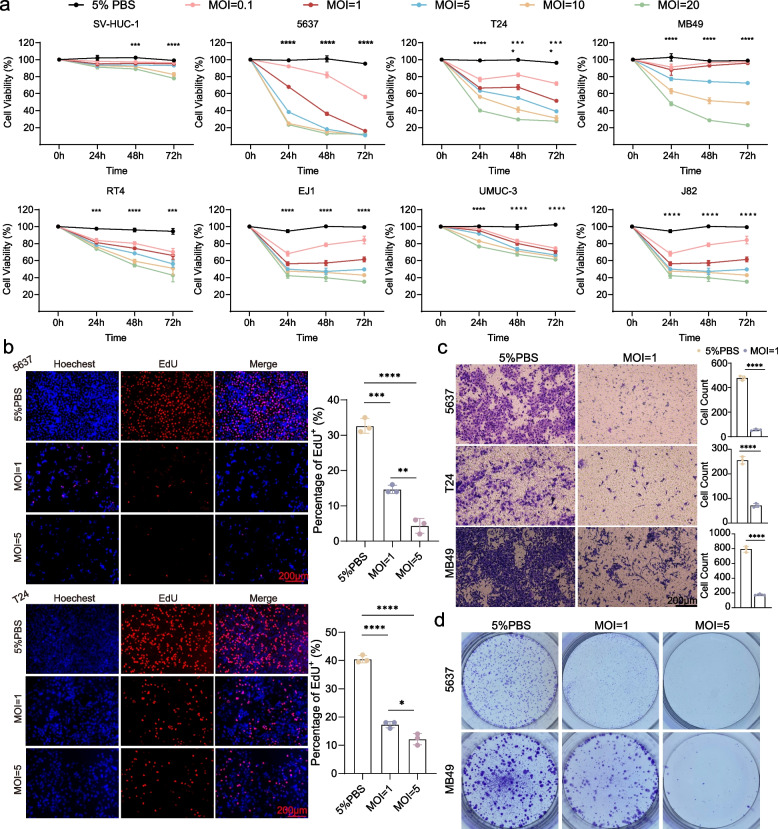
In vitro validation of anti-tumor effect of OHSV2 in bladder cancer. **a** The viability of different bladder cancer cell lines treated with different concentrations of oncolytic virus (5% PBS, MOI = 0.1, MOI = 1, MOI = 5, MOI = 10, MOI = 20) (n = 6). **b** EdU assay for proliferation of 5637 and T24 treated with different concentrations (5% PBS, MOI = 1, MOI = 5) (n = 3). **c** Cell migration experiment to assess the impact of oncolytic virus treatment (5% PBS, MOI = 1) on the migration ability of different bladder cancer cell lines (n = 3). **d** Changes in the clonogenic ability of bladder cancer cells after treatment with different concentrations of oncolytic virus (5% PBS, MOI = 1, MOI = 5). Data are presented as mean ± SD. Statistical significance was determined by unpaired two-tailed Student's t-test (*p < 0.05, **p < 0.01, ***p < 0.001, ****p < 0.0001)

**Fig. 2:**
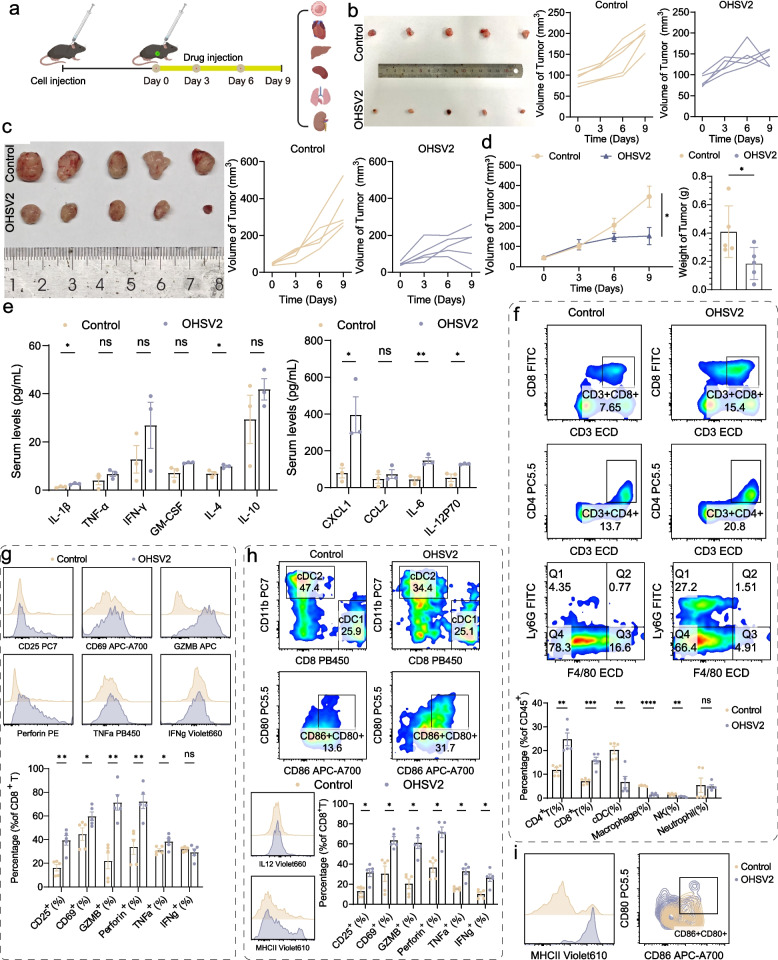
OHSV2 suppresses tumor growth and activates antitumor immunity in vivo. **a** Schematic diagram for in vivo validation of the antitumor efficacy of OHSV2 in a bladder cancer subcutaneous xenograft tumor model. **b** Ex vivo images of tumors after BALB/c Nude mouse sacrifice and tumor growth curve. **c** Ex vivo images of tumors after C57BL/6 mouse sacrifice and tumor growth curve. **d** Line graph showing changes in tumor volume during the treatment period (n = 5). **e** Multiplex cytokine analysis of mouse serum(n = 3). **f** Flow cytometry analysis of tumor-infiltrating immune cells (n = 5). **g** Functional analysis of T cells. Expression of CD25, CD69, granzyme B (GZMB), perforin, and tumor necrosis factor-α (TNF-α) was upregulated (n = 5). **h** Functional analysis of dendritic cells (DCs). Expression of CD80, CD86, major histocompatibility complex class II (MHC-II), and interleukin-12 (IL-12) was upregulated (n = 5). **i** Macrophage function analysis (n = 5). Data are presented as mean ± SD. Statistical significance was determined by unpaired two-tailed Student's t-test (*p < 0.05, **p < 0.01, ***p < 0.001, ****p < 0.0001)

To evaluate the direct and systemic anti-tumor effects of OHSV2 against bladder cancer, we conducted a series of in vitro and in vivo experiments. The results of Cell Counting Kit-8 (CCK-8) assay showed that viability of both human and murine bladder cancer cell lines decreased in a dose/time-dependent manner with OHSV2 treatment at the indicated multiplicity of infection (MOI)(Fig. 1a, Fig. S1a). We selected representative human (5637, T24) and murine (MB49) bladder cancer cell lines for subsequent research, based on their common use as experimental models in bladder cancer research. EdU staining results revealed that, compared with control group (5% PBS), the 5-ethynyl-2′-deoxyuridine (EdU) staining in the OHSV2-treated group was significantly reduced (Fig. 1b). The results of cell migration experiment showed that OHSV2 significantly weakened migration ability of bladder cancer cells (Fig. 1c). After oncolytic virus treatment, the number of cell colonies in all three cell lines was significantly reduced, indicating that OHSV2 significantly weakened the cells' clonogenic ability (Fig. 1d). We simultaneously detected the infection of bladder cancer cells by OHSV2 through the expression of GFP and transcription levels of granulocyte–macrophage colony-stimulating factor (GM-CSF) genes (Fig. S1b, c). To investigate the in vivo antitumor efficacy of the oncolytic virus, we established subcutaneous xenograft tumor models in C57BL/6 mice derived from MB49 cells and in BALB/c-Nude mice derived from T24 cells (Fig. 2a). We found that the subcutaneous tumor volume in the treatment group was significantly smaller than that in control group (Fig. 2b, c and Fig. S2a). Upon measurement and calculation, the ex vivo tumor volume in the OHSV2 group was significantly lower than that in control group (Fig. S2b). The tumor weight in the OHSV2 group was also significantly lower than that in control group (Fig. 2d, and Fig. S2d). Additionally, there was no significant change in body weight between the OHSV2 and control groups (Fig. S2c). Hematoxylin and eosin (H&E) staining of vital organs from both the OHSV2-treated and control mice revealed no obvious abnormalities or significant differences in the structure of these organs, indicating the safety of in vivo oncolytic virus therapy (Fig. S2e and Fig. S3a). To further investigate the impact of oncolytic virus on the immune system, we performed multiplex cytokine analysis. We found that serum levels of interleukin-1β (IL-1β), C-X-C motif chemokine ligand 1 (CXCL1), interleukin-6 (IL-6), and interleukin-12 (IL-12) were significantly upregulated following OHSV2 treatment (Fig. 2e and Fig. S3b). To characterize the changes in the tumor immune microenvironment, we performed flow cytometry on tumor tissues (Fig. S3c, d). We found that infiltration of both CD4+ and CD8+ T cells was significantly increased, whereas the proportions of dendritic cells (DCs), macrophages, and natural killer (NK) cells, were decreased (Fig. 2f). To further assess the functional status of T cells and DCs, we performed additional analyses. We observed significant activation of T cells, as evidenced by upregulation of CD25, CD69, granzyme B (GZMB), perforin, and tumor necrosis factor-α (TNF-α) (Fig. 2g). Similarly, DCs showed enhanced maturation, with increased expression of CD80, CD86, major histocompatibility complex class II (MHC-II), and IL-12 (Fig. 2h). Macrophages also exhibited upregulation of MHC-II, indicating enhanced antigen-presenting function (Fig. 2i). We also measured liver and kidney function in mice and found that intratumoral injection of OHSV2 did not significantly affect these parameters (Fig. S3e). In summary, OHSV2 demonstrates potent anti-tumor activity against bladder cancer in vitro and in vivo, coupled with a favorable safety profile and the capacity to stimulate anti-tumor immunity.

### OHSV2 induces pyroptosis in bladder cancer through canonical pathway

**Fig. 3:**
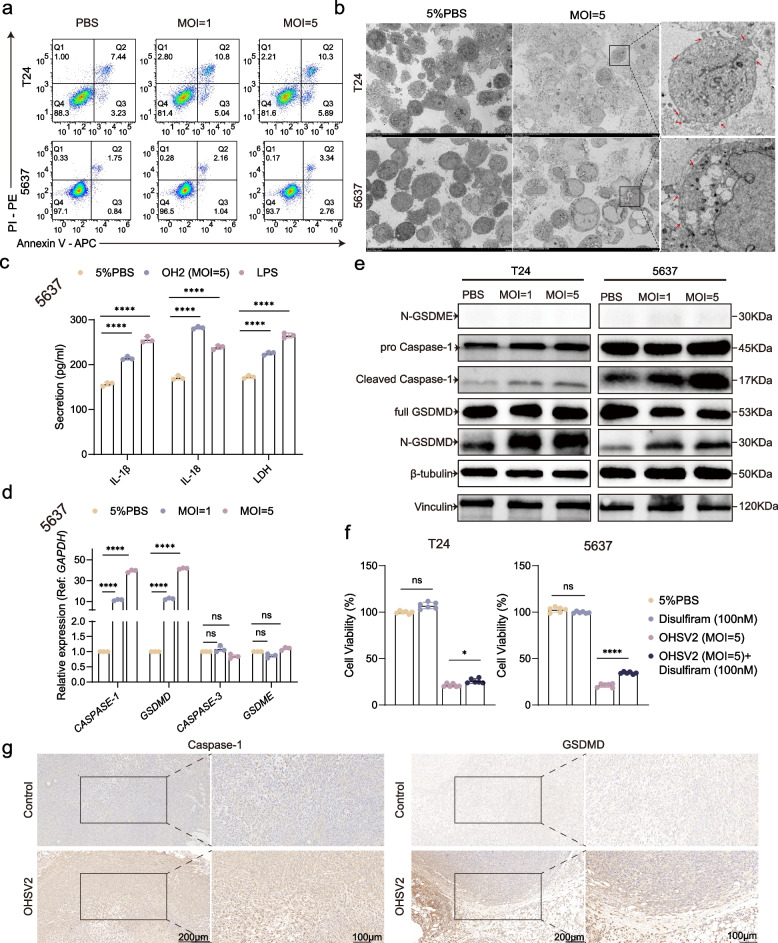
OHSV2 induces pyroptosis in bladder cancer via the Caspase-1/GSDMD canonical pathway. **a** Flow cytometry analysis of apoptosis rates in bladder cancer cells treated with different concentrations of OHSV2 (5% PBS, MOI = 1, MOI = 5). **b** Transmission electron microscopy observation of morphological changes in bladder cancer cells under different OHSV2 treatments (5% PBS, MOI = 5). The red arrows indicate typical pyroptosis morphology. **c** Expression levels of IL-18, IL-1β, and LDH in the cell supernatant after OHSV2 treatment of 5637 cells (n = 3). **d** RT-qPCR analysis of the mRNA levels of *CASP1, GSDMD, CASP3, GSDME* in 5637 cells treated with different concentrations (5% PBS, MOI = 1, MOI = 5) (n = 3). **e** Western Blot analysis of the expression levels of Caspase-1, GSDMD and GSDME in bladder cancer cells treated with different concentrations (5% PBS, MOI = 1, MOI = 5). **f** CCK-8 assay evaluating cell viability after OHSV2 treatment with the pyroptosis inhibitor Disulfiram (n = 6). **g** Immunohistochemistry analysis to detect the expression of Cleaved Caspase-1 and N-GSDMD proteins in subcutaneous xenograft tumor tissues from nude mice. Data are presented as mean ± SD. Statistical significance was determined by unpaired two-tailed Student's t-test (*p < 0.05, **p < 0.01, ***p < 0.001, ****p < 0.0001)

To elucidate the mechanism of cell death induced by OHSV2, we investigated whether pyroptosis was involved. Apoptosis flow cytometry indicated that OHSV2 could induce apoptosis in bladder cancer cells (Fig. 3a). However, upon comparison with the results of CCK-8 assay, it was found that the proportion of apoptotic cells was lower than the total proportion of cell death (Fig. S4b). Therefore, we hypothesized that in addition to apoptosis, other modes of cell death occurred following OHSV2 treatment. Under optical microscope, we observed that after OHSV2 treatment, some cells underwent morphological changes characterized by cell swelling and enlargement, with many bubble-like protrusions on cell membrane surface (Fig. S4a). These specific morphological changes are consistent with those of pyroptosis. First, we used transmission electron microscopy to observe cell morphology. In OHSV2-treated group, some cells were swollen and vacuolated, with small vesicles protruding from cell membrane, presenting morphological characteristics of pyroptosis (Fig. 3b). Subsequently, we detected the levels of interleukin-18 (IL-18), interleukin-1β (IL-1β), and lactate dehydrogenase (LDH) in the cell supernatant using enzyme-linked immunosorbent assay (ELISA). Using the 5637 cell line as a representative model, we found that after OHSV2 treatment, production of IL-18, IL-1β, and LDH increased (Fig. 3c), with consistent results observed in T24 cells (Fig. S4c). Above results suggest that oncolytic virus OHSV2 can induce pyroptosis in bladder cancer cells. To investigate whether oncolytic virus OHSV2 induces pyroptosis in bladder cancer cells through the canonical or non-canonical pathway, we further detected the expression of Caspase-1, GSDMD, Caspase-3, and Gasdermin E (GSDME) at both RNA and protein levels. RT-qPCR results in 5637 cells showed that after OHSV2 treatment, RNA expression levels of Caspase-1 and GSDMD were significantly upregulated, while RNA levels of Caspase-3 and GSDME remained unchanged (Fig. 3d). Parallel experiments in T24 cells yielded similar results (Fig. S4d). Consistent with this, Western Blot results demonstrated a significant increase in the expression of Cleaved Caspase-1 and N-GSDMD, while N-GSDME was not expressed in either control or treatment group (Fig. 3e). The use of GSDMD inhibitor Disulfiram can partially reverse decrease in bladder cancer cell viability caused by OHSV2 (Fig. 3f). Additionally, we collected tumor tissues from nude mice for Western Blot and immunohistochemical analysis. We found that in the oncolytic virus-treated group, the expression of Cleaved Caspase-1 and N-GSDMD in the tumor tissues of the nude mice was significantly increased (Fig. 3g, and Fig. S4e). Thus, we have found that OHSV2 treatment of bladder cancer cells induces pyroptosis in these cells through the canonical Caspase-1/GSDMD pathway.

### NLRP3 is the key upstream component in pyroptosis induced by OHSV2

**Fig. 4:**
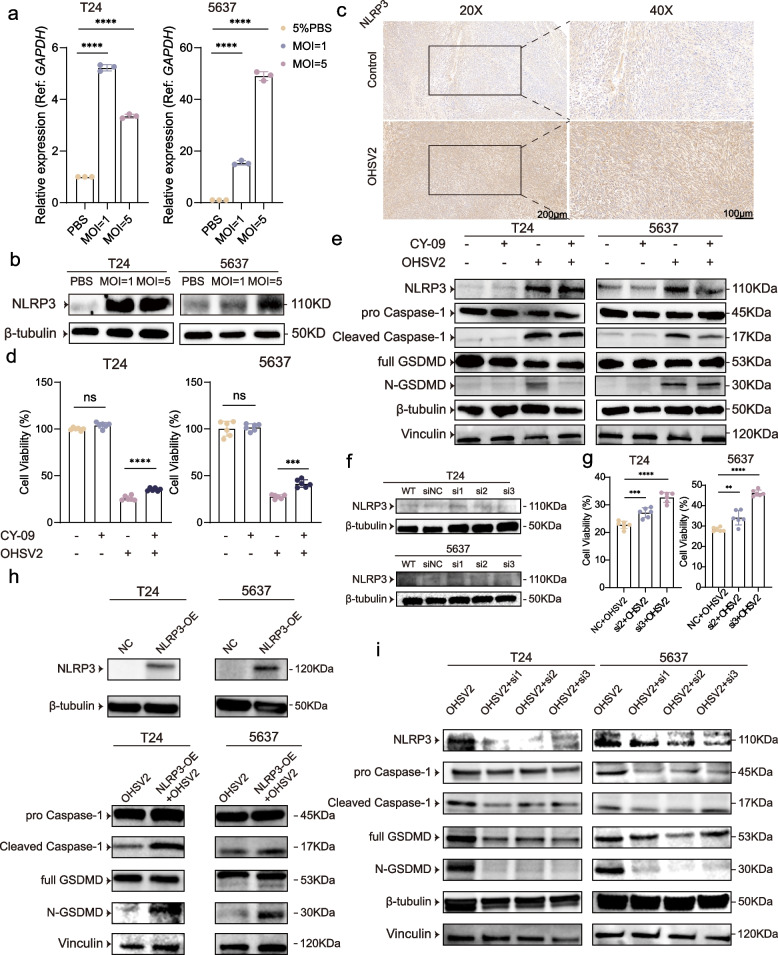
NLRP3 is the key upstream component in pyroptosis induced by OHSV2. **a** RT-qPCR analysis of the expression levels of *NLRP3* in bladder cancer cells treated with different concentrations (5% PBS, MOI = 1, MOI = 5) (n = 3). **b** Western Blot analysis of the expression levels of NLRP3 in bladder cancer cells treated with different concentrations (5% PBS, MOI = 1, MOI = 5). **c** Immunohistochemistry analysis to detect the expression of NLRP3 proteins in subcutaneous xenograft tumor tissues from nude mice. **d** CCK-8 assay to evaluate the effects of OHSV2 in combination with an NLRP3 inhibitor (CY-09) on the viability of bladder cancer cells (n = 6). **e** Western Blot analysis to evaluate the effects of OHSV2 in combination with an NLRP3 inhibitor (CY-09) on the expression of NLRP3, Caspase-1, and GSDMD. **f** Western blot analysis of NLRP3 after NLRP3 knockdown. **g** CCK-8 assay showing partial restoration of cell viability after NLRP3 knockdown (n = 6). **h** Western blot analysis of NLRP3, Cleaved Caspase-1, and N-GSDMD expression in NLRP3-overexpressing cells after OHSV2 treatment. **i** Western blot analysis of NLRP3, Cleaved Caspase-1, and N-GSDMD expression in NLRP3-knockdown cells after OHSV2 treatment. Data are presented as mean ± SD. Statistical significance was determined by unpaired two-tailed Student's t-test (*p < 0.05, **p < 0.01, ***p < 0.001, ****p < 0.0001)

We next sought to identify the upstream pattern recognition receptor responsible for Caspase-1 activation. Previous studies have shown that the activation and cleavage of Caspase-1 in the canonical pyroptosis pathway depend on the activation of upstream pattern recognition receptors (PRRs). NOD-like receptor family pyrin domain containing 3 (NLRP3) is a classical upstream PRR. To determine whether oncolytic virus OHSV2 activates NLRP3, we detected RNA and protein expression levels of NLRP3. We found that OHSV2 treatment significantly upregulates the expression of NLRP3 in bladder cancer cell lines (Fig. 4a, b). Additionally, Western Blot and immunohistochemical staining results showed that NLRP3 was activated in tumor tissues following intratumoral injection of OHSV2 in the subcutaneous xenograft tumor model of mice (Fig. 4c, and Fig. S5a). To verify whether the activation of Caspase-1/GSDMD pathway depends on the activation of NLRP3, we combined oncolytic virus OHSV2 with NLRP3 inhibitor CY-09. The results showed that CY-09 could partially restore the viability of bladder cancer cells (Fig. 4d). Furthermore, after combining CY-09, the activation of NLRP3 was inhibited, and the activation of downstream Caspase-1/GSDMD was also inhibited (Fig. 4e). To further confirm the central role of NLRP3 in OHSV2-induced pyroptosis, we established NLRP3-knockdown cell lines (Fig. 4f). We found that cell viability was partially restored after NLRP3 knockdown (Fig. 4g). Furthermore, we examined the activation of the pathway. NLRP3-overexpressing bladder cancer cells exhibited stronger pyroptosis upon OHSV2 treatment (Fig. 4h), whereas NLRP3 knockdown reversed OHSV2-induced pyroptosis (Fig. 4i).

### OHSV2 induces NLRP3/Caspase-1/GSDMD-dependent pyroptosis in bladder cancer cells via TLR4

**Fig. 5:**
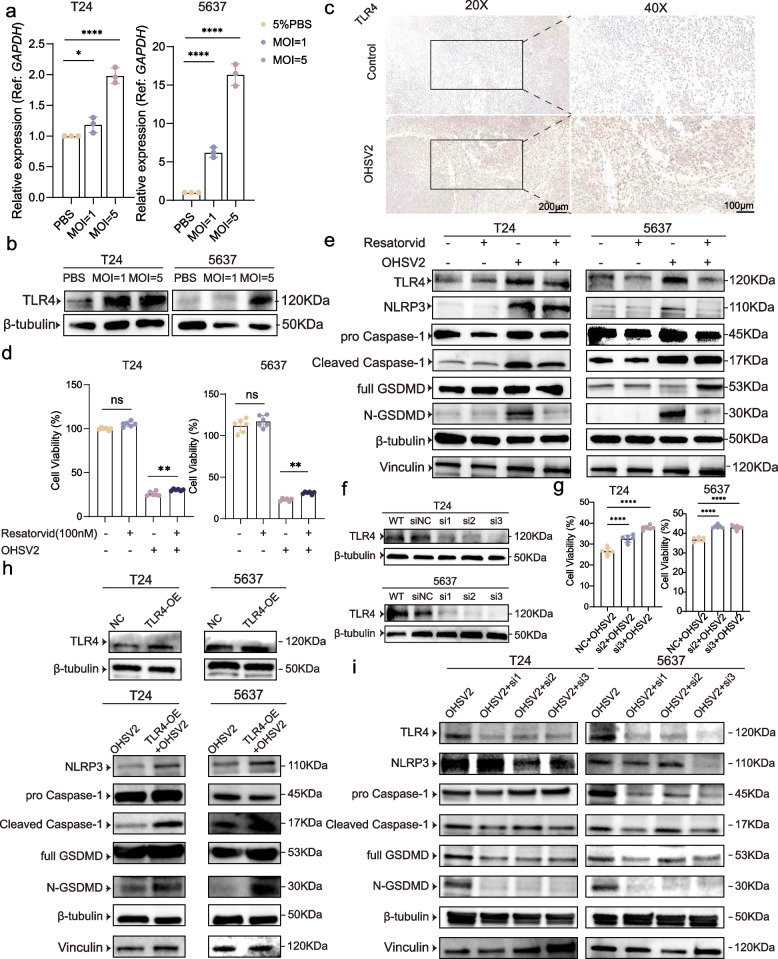
OHSV2 induces NLRP3/Caspase-1/GSDMD-dependent pyroptosis in bladder cancer cells via TLR4. **a** RT-qPCR analysis of the expression levels of *TLR4* in bladder cancer cells treated with different concentrations (5% PBS, MOI = 1, MOI = 5) (n = 3). **b** Western Blot analysis of the expression levels of TLR4 in bladder cancer cells treated with different concentrations (5% PBS, MOI = 1, MOI = 5). **c** Immunohistochemistry analysis to detect the expression of TLR4 proteins in subcutaneous xenograft tumor tissues from nude mice. **d** CCK-8 assay to evaluate the effects of OHSV2 in combination with an TLR4 inhibitor (Resatorvid) on the viability of bladder cancer cells (n = 6). **e** Western Blot analysis to evaluate the effects of OHSV2 in combination with an TLR4 inhibitor (Resatorvid) on the expression of TLR4, NLRP3, Caspase-1, and GSDMD. **f** Western blot analysis of TLR4 after TLR4 knockdown. **g** CCK-8 assay showing partial restoration of cell viability after TLR4 knockdown (n = 6). **h** Western blot analysis of NLRP3, Cleaved Caspase-1, and N-GSDMD expression in TLR4-overexpressing cells after OHSV2 treatment. **i** Western blot analysis of NLRP3, Cleaved Caspase-1, and N-GSDMD expression in TLR4-knockdown cells after OHSV2 treatment. Data are presented as mean ± SD. Statistical significance was determined by unpaired two-tailed Student's t-test (*p < 0.05, **p < 0.01, ***p < 0.001, ****p < 0.0001)

Having established NLRP3 as the key upstream sensor, we sought to identify the trigger for its activation. As viral components are known to be sensed by pattern recognition receptors such as Toll-like receptors (TLRs), and given that Toll-like receptor 4 (TLR4) signaling is a canonical upstream pathway for NLRP3 inflammasome activation, we investigated whether TLR4 mediated the pyroptotic effect of OHSV2 [9, 10]. We detected the RNA and protein expression levels of TLR4. We found that OHSV2 treatment significantly upregulated the expression of TLR4 (Fig. 5a, b). Additionally, Western Blot and immunohistochemical staining results showed that TLR4 was upregulated in tumor tissues following intratumoral injection of OHSV2 (Fig. 5c and Fig. S6 a, b). The use of TLR4 inhibitor Resatorvid could significantly restore viability of bladder cancer cells (Fig. 5d). Moreover, co-treatment with Resatorvid and oncolytic virus downregulated this pyroptosis pathway (Fig. 5e). To further clarify the critical role of TLR4 in this process, we established TLR4-knockdown and TLR4-knockout cell lines (Fig. 5f, Fig. S6c-e). We found that cell viability was partially restored in the knockdown cells following OHSV2 treatment (Fig. 5g). We then examined the activation of the pyroptosis pathway in these cells upon OHSV2 stimulation. The results showed that TLR4-overexpressing bladder cancer cells exhibited stronger pathway activation after OHSV2 treatment (Fig. 5h), whereas TLR4 knockdown or knockout significantly attenuated OHSV2-induced pathway activation (Fig. 5i and Fig. S6f). CCK-8 assay showed that QNZ partially reversed OHSV2-induced cell death (Fig. S6g), suggesting that nuclear factor kappa-light-chain-enhancer of activated B cells (NF-κB) signaling is required for TLR4-mediated pyroptosis. To contextualize our findings, we performed bioinformatic analyses of public datasets from other cancer models treated with HSV-based oncolytic viruses (Fig. S8). While pyroptosis-related genes were frequently induced, the significant upregulation of TLR4 observed in our bladder cancer models was not a universal feature across all tumor types.

### Combining TLR4 agonists with OHSV2 enhances antitumor efficacy by amplifying pyroptosis and immune activation

**Fig. 6:**
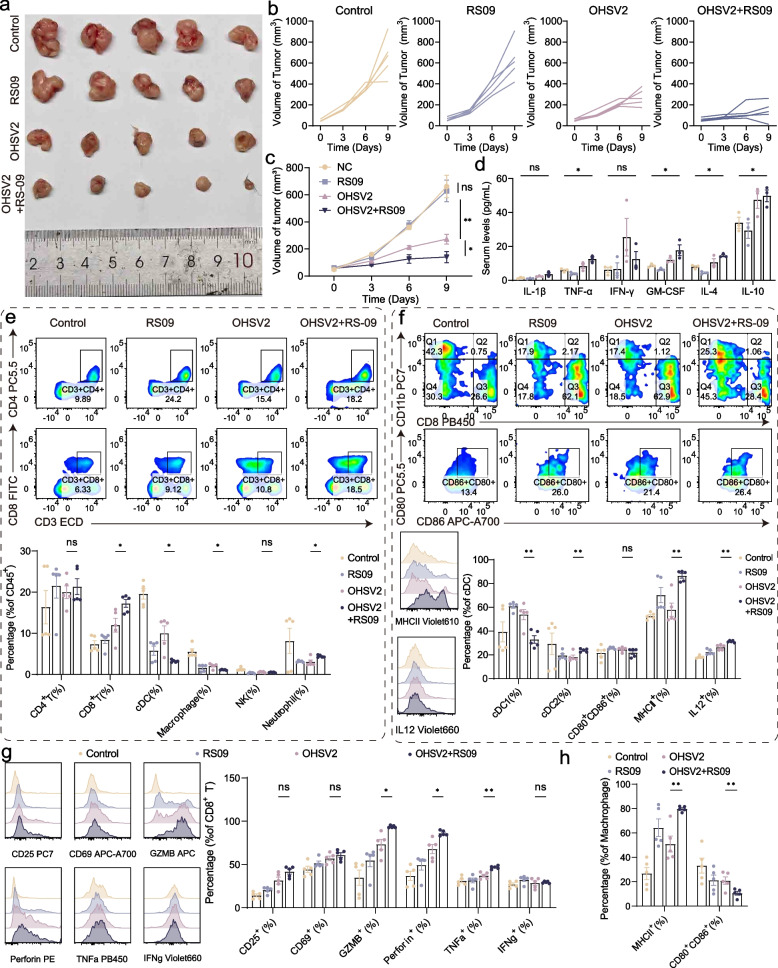
Combination of RS-09 and OHSV2 can increase the level of pyroptosis in a bladder cancer subcutaneous xenograft tumor model. **a** Images of excised tumors. **b**-**c** Tumor growth curves (n = 5). **d** Multiplex cytokine analysis of mouse serum (n = 5). **e** Flow cytometry analysis of tumor-infiltrating T cells (n = 5). **f** Functional analysis of dendritic cells (n = 5). **g** Functional analysis of T cells (n = 5). **h** Macrophage function analysis (n = 5). Data are presented as mean ± SD. Statistical significance was determined by unpaired two-tailed Student's t-test (*p < 0.05, **p < 0.01, ***p < 0.001, ****p < 0.0001)

Given the central role of TLR4, we explored the therapeutic potential of combining OHSV2 with TLR4 pathway agonists. We further evaluated the combination of OHSV2 with the TLR4 agonist RS-09 in vivo. The combination treatment showed significant tumor control (Fig. 6a-c). Multiplex cytokine analysis of mouse serum revealed that TNF-α and GM-CSF were significantly upregulated in the combination group (Fig. 6d, Fig. S7a). To further investigate the impact on the tumor immune microenvironment, we performed flow cytometry on tumor tissues (Fig. S7b). We observed increased infiltration of CD3^+^CD8^+^ T cells, whereas no significant change was detected in CD4^+^ T cells (Fig. 6e). Functional analysis showed that although DCs did not exhibit significantly elevated CD80 or CD86 expression, MHC-II and IL-12 were significantly upregulated (Fig. 6f). T cells also showed enhanced effector function, with upregulation of granzyme B, perforin, and TNF-α (Fig. 6g). Macrophages displayed increased MHC-II expression (Fig. 6h). No significant changes in serum liver and kidney function were observed in the combination group (Fig. S7c). To contextualize our findings, we performed bioinformatic analyses of public datasets from other cancer models treated with HSV-based oncolytic viruses (Fig. S8). Immunohistochemical analysis further confirmed that the TLR4/NLRP3/Caspase-1/GSDMD pathway was significantly activated in the combination group (Fig. S9). Bacillus Calmette-Guérin (BCG) is one of the standard treatments for bladder cancer and has also been found to be an agonist of TLR4 [11, 12]. Therefore, we combined BCG and OHSV2 in a subcutaneous xenograft tumor model in nude mice. We found that intratumoral injection of BCG had a certain effect on bladder cancer control (Fig. S10a). Compared with oncolytic virus monotherapy group, the tumor volume and weight in combination therapy group were lower, although there was no significant statistical difference (Fig. S10b, c). Furthermore, we performed immunohistochemical staining on the tumor tissues after intervention and found that the TLR4/NLRP3/Cleaved Caspase-1/N-GSDMD pathway was significantly upregulated in the combination group, indicating a stronger level of pyroptosis (Fig. S10d). These findings collectively demonstrate that co-targeting the TLR4 pathway enhances the anti-tumor efficacy of OHSV2 by amplifying pyroptosis and promoting a more robust anti-tumor immune response.

## Discussion

This study evaluated the oncolytic efficacy of OHSV2 against bladder cancer. OHSV2 is a distinct clinical-stage candidate with several advantageous features. It is built on a herpes simplex virus type 2 (HSV-2) backbone, which possesses a large genome accommodating substantial genetic engineering [13]. Notably, compared to HSV-1-based vectors, HSV-2 may exhibit a distinct infection dynamic, where a potentially more rapid onset of cytotoxicity could offer a therapeutic advantage, despite a typically lower viral burst size [14]. The construct is further safely attenuated by deleting the neurovirulence gene ICP34.5 and the immune evasion gene ICP47, and it is armed with GM-CSF to potentiate antitumor immunity. Its promising clinical translation is evidenced by key regulatory endorsements, including Orphan Drug Designation (ODD) and Fast Track Designation (FTD) from the U.S. FDA. Our results confirm its potent, dose-dependent oncolytic activity against bladder cancer cells, consistent with its established preclinical efficacy and favorable safety profile in other models and early-phase trials [15].

However, only four patients achieved partial remission in the aforementioned clinical trial after initial treatment. This discrepancy between robust preclinical efficacy and modest clinical outcomes suggests that human pathophysiology may present greater complexity than cellular or animal models, and the mechanistic basis of OHSV2's therapeutic effects remains incompletely understood. Therefore, elucidating the molecular mechanisms underlying OHSV2's antitumor activity is critical to enhancing its clinical performance. Wu et al. performed single-cell RNA sequencing on peripheral blood from CT26 subcutaneous tumor-bearing mice following intratumoral OHSV2 injection, revealing systemic immune activation characterized by elevated cytotoxic CD8 + T cells and mature NK cells [16]. Kong et al. further demonstrated OHSV2's role in promoting M1 macrophage polarization to augment antitumor immunity [17].

In this study, we identified that OHSV2 induces pyroptosis in bladder cancer cells via the canonical Caspase-1/Gasdermin D (GSDMD) pathway. Pyroptosis, a lytic programmed cell death mechanism, plays an important role in innate immunity, inflammation, host defense, and tumor modulation [18]. Analogous to our findings, Victorio et al. reported that Zika virus (ZIKV-LAV) triggered glioma cell pyroptosis through GSDMD cleavage [19], while Wu et al. observed that recombinant measles virus (rMV-Hu191) induced pyroptosis in esophageal squamous cell carcinoma via the Caspase-3/GSDME axis, dependent on BCL2 antagonist/killer (BAK)/BCL2-associated X protein (BAX)-mediated mitochondrial dysfunction [20]. Notably, our data excluded Caspase-3/GSDME pathway involvement in OHSV2-induced pyroptosis, confirming its reliance on the canonical route.

Moreover, we demonstrated that OHSV2 robustly activates NLRP3 in bladder cancer cells. NLRP3 inhibition not only restored cell viability but also abrogated Caspase-1/GSDMD-mediated pyroptosis, positioning NLRP3 as the key pattern recognition receptor (PRR) in this cascade. Consistent with this, Wang and Choudhury et al. respectively implicated NLRP3 in interleukin-1β (IL-1β) secretion triggered by Newcastle disease virus and Seneca Valley virus [21, 22]. These findings suggest NLRP3-targeted therapies may potentiate oncolytic virotherapy. However, whether additional PRRs contribute to OHSV2's mechanism requires further investigation.

We further uncovered TLR4's role in mediating NLRP3/Caspase-1/GSDMD-dependent pyroptosis to enhance OHSV2's cytotoxicity. Earlier studies by Georgel et al. and Liu et al. documented TLR4 activation by vesicular stomatitis virus and HSV-2 [23], respectively. Although TLR4 is generally downregulated in bladder cancer [24, 25], its activation exhibits dualistic effects: Zhang et al. reported that TLR4/CD14 upregulation by Polyporus polysaccharide-BCG combination therapy suppressed tumor invasiveness [26], whereas Wang et al. found TLR4-mediated B7-H1 expression shielded T24 cells from cytotoxic T lymphocyte (CTL) attack [27]. This functional dichotomy may depend on agonist dosage [27]. The critical role of TLR4 was demonstrated both pharmacologically, using the specific inhibitor Resatorvid which blocks intracellular signal transduction, and genetically, via knockdown and overexpression studies, confirming its position as the upstream sensor initiating OHSV2-induced pyroptosis.

It is important to note that the interaction between TLR4 and NLRP3 is likely indirect, mediated by intracellular signaling cascades. While our study demonstrates the functional requirement of TLR4 and implicates NF-κB signaling, future studies are warranted to map the precise molecular events connecting these key players [28]. Notably, BCG—the first-line intravesical therapy for NMIBC—acts as a TLR4 agonist, initiating the myeloid differentiation primary response 88 (MyD88)/NF-κB pathway to drive cytokine production and immune activation [29–31]. Reis et al. demonstrated superior efficacy of combined TLR4 activation (via castration) and BCG in a rat orthotopic model [32]. In our final experiment, while OHSV2-BCG coadministration in nude mouse xenografts showed only a trend toward improved outcomes (statistically nonsignificant, potentially due to limited sample size), it distinctly amplified TLR4/NLRP3/Caspase-1/GSDMD signaling. Given pyroptosis's immunogenic nature, its full therapeutic potential may require intact host immunity, suggesting combinatorial strategies with TLR4 agonists could benefit OHSV2-refractory patients [33]. Notably, the central role of TLR4 may be context-specific. Bioinformatic analysis of other tumor models treated with HSV-based oncolytic viruses revealed that while pyroptosis execution is a common outcome, a consistent and significant upregulation of TLR4 is not universally observed. This contrast underscores that the robust TLR4/NLRP3/GSDMD axis we define here may be a particularly salient mechanism in bladder cancer, possibly influenced by the unique tumor microenvironment. This specificity informs the translational rationale for targeting this pathway in bladder cancer with OHSV2 [34].

This study has several limitations that should be acknowledged. While our subcutaneous xenograft models established proof-of-concept efficacy and mechanism, they do not fully recapitulate the orthotopic bladder tumor microenvironment or the clinically relevant route of intravesical instillation. Future studies employing orthotopic models or spontaneous canine models of bladder cancer (which allow for cystoscopic evaluation and instillation) will be critical to validate the therapeutic potential and safety of OHSV2, particularly in combination with agents like BCG, via intravesical delivery [35]. While our functional experiments, including the use of an NF-κB inhibitor, confirm the essential role of TLR4 signaling in NLRP3 inflammasome activation, the specific signaling details—such as how the MyD88/TIR-domain-containing adapter-inducing interferon-β (TRIF) adaptor proteins downstream of TLR4 precisely regulate NLRP3 expression and assembly remain incompletely elucidated. The evidence from this study supports the canonical model wherein TLR4 indirectly regulates NLRP3 via downstream signaling cascades (e.g., the NF-κB pathway). However, whether OHSV2 might also engage additional TLR4-independent pathways to initiate inflammasome activation awaits further investigation. These knowledge gaps highlight the need for future studies employing more physiologically relevant models, comprehensive pathway analyses, and detailed mechanistic investigations to fully elucidate OHSV2's therapeutic potential in bladder cancer.

Our study is the first to delineate the TLR4/NLRP3/Caspase-1/GSDMD pyroptosis axis in OHSV2-treated bladder cancer, which distinguishes its mechanism from other oncolytic viruses. Therefore, the major translational insight of this work is the identification of a novel, targetable cell death pathway that addresses a central challenge in oncolytic virotherapy—modest monotherapy efficacy. By elucidating the TLR4-dependent pyroptosis mechanism, our study provides a clear rationale for combining OHSV2 with immunomodulators to enhance antitumor immunity and improve clinical outcomes, charting a concrete path for its future development in bladder cancer.

## Materials and methods

### Oncolytic virus

OHSV2 was provided by Binhui Biopharmaceutical Co., Ltd. (Wuhan, China). This virus is based on the HSV-2 HG52 strain and has been engineered by deleting the ICP47 and ICP34.5 genes to reduce neurotoxicity to normal cells and diminish its ability to evade the immune system, as described for the oHSV2 backbone [36]. Additionally, the gene encoding human granulocyte–macrophage colony-stimulating factor (GM-CSF), CSF2, was inserted into the virus to enhance the recruitment and activation of antigen-presenting cells, a strategy established to potentiate the immunotherapeutic effect of oncolytic vectors [37]. The Multiplicity of Infection (MOI) for the oncolytic virus OHSV2 is calculated based on the specific experimental setup and is given by the formula:

$$MOI = \frac{0.7 \times Virus\ concentration\ titer \times Virus\ volume}{Number\ of\ cells}$$

### Cell culture

SV-HUC-1, 5637, T24, RT4, UMUC-3, J82, and EJ1 cells were cultured in complete medium composed of 1640 medium (Gibco, Rockville, USA) + 10% fetal bovine serum (FBS, ABW, Shanghai, China) + 1% penicillin–streptomycin-amphotericin B solution (Procell, Wuhan, China). The MB49 cells were cultured in complete medium composed of high-glucose DMEM medium (Gibco, Rockville, USA) + 10% FBS +1% penicillin–streptomycin-amphotericin B solution (Procell, Wuhan, China). The culture conditions were maintained at 37 °C with 5% CO₂ in a cell incubator. The cells were observed under an optical microscope daily, and the medium was changed and cells were passaged in a timely manner. For in vitro pharmacological interventions, the following inhibitors were used at optimized concentrations based on preliminary dose–response experiments in bladder cancer cell lines: Disulfiram (GSDMD inhibitor, 100 nM), CY-09 (NLRP3 inhibitor, 100 nM), Resatorvid (TLR4 inhibitor, 100 nM), and QNZ (NF-κB inhibitor, 100 nM). All inhibitors were purchased from MedChemExpress (MCE, USA).

### Mice

Male C57BL/6 mice and BALB/c nude mice aged 4–6 weeks (purchased from Beijing Vital River Co., Ltd) were selected for in vivo tumor growth experiments. All animal experiments were conducted in a Specific Pathogen-Free (SPF) laminar flow animal laboratory. After purchase, the experimental animals were acclimatized and observed for one week before the commencement of relevant experimental procedures. This study primarily established two types of subcutaneous xenograft tumor models in mice. All experimental procedures strictly adhered to the national and institutional regulations for laboratory animal management and were approved by the Experimental Animal Ethics Committee of Tongji Hospital (approval number: TJH-202211020).

### CCK-8 assay

Rinse each well of the 96 well plate with 100 µL Phosphate-Buffered Saline (PBS, Procell, China) and add an equal amount of PBS to the peripheral wells to reduce volatilization. Subsequently, the cells were digested with trypsin containing 0.25% ethylenediaminetetraacetic acid (EDTA) (Procell, China), and digestion was terminated using a culture medium containing fetal bovine serum to prepare a cell suspension. After counting and adjusting the concentration, 7000 cells per well (100 µL suspension) were seeded onto a 96 well plate and cultured for 12 h to allow the cells to adhere to the wall. Discard the culture medium before intervention, add intervention solution and continue to culture for the corresponding time. Fresh CCK-8 working solution (ABclonal, China) was added to each well. After incubation for 2 h, the OD value at 450 nm was measured using a microplate reader. Cell viability was calculated using the formula:

$$Cell \, viability = \frac{Experimental \, group \, OD-Blank \, group \, OD}{Control \, group \, OD-Blank \, group \, OD}\times \mathrm{100}\%$$

where the blank group represents wells without cells.

### 5-Ethynyl-2'-deoxyuridine (EdU) staining

The digested cell suspension was counted and then seeded into 6-well plates at a density of 500, 000 cells per well, cultured for 12 h to make it adhere to the wall, and then cultured for another 24 h after corresponding intervention treatment.

In the experiment, the 10 mm EdU stock solution (Beyotime, China) should be diluted into 20 µM 2 × working solution with the culture medium at the ratio of 1:500. After preheating, it should be mixed with the same amount of culture medium and added to the well plate to make the final concentration reach 10 µM, and the label should be incubated for 2 h.

After labeling, the cells were washed with PBS, fixed with 4% paraformaldehyde (Beyotime, China) for 15 min, then washed three times with PBS (4 min each time), permeabilized with 0.3%tritonx-100 (Beyotime, China) for 10–15 min, and washed again. After that, 0.5 mL of the prepared click reaction solution was added to each well to avoid light for 30 min.

### Cell migration assay

After the corresponding treatments, cells were cultured for an additional 12 h. Subsequently, the cells were digested with trypsin containing 0.25% EDTA, and were resuspended in serum-free medium. The cell concentration was adjusted to 80,000 cells per 200 µL.

In a 24-well plate, 600 µL of culture medium containing 20% FBS was added to each well. The Transwell insert was placed into the well, and 200 µL of the cell suspension was added to the upper chamber. The cells were cultured at 37 °C with 5% CO₂ for 48 h. After the culture period, the insert was removed, the culture medium was discarded, and the cells were fixed with 4% formaldehyde for 30 min. The cells were then stained with crystal violet (Beyotime, China) for 30 min. After washing with ddH₂O to remove residual stain, the non-migrated cells on the upper surface of the insert were gently removed with a cotton swab. The insert was air-dried, and the migrated cells were observed and counted under a microscope.

### Colony formation assay

After the corresponding treatments, the cells were cultured for an additional 12 h. Subsequently, the cells were digested again, resuspended, and adjusted to a concentration of 1,000 cells per well. They were then seeded into a 6-well plate and supplemented with 2 mL of culture medium. The cells were cultured statically for 14 days (avoiding movement of the culture plate during this period). Once visible cell colonies had formed, the culture was terminated. The culture medium was discarded, and the cells were gently washed with PBS to remove cell debris. One milliliter of fixative was added to cover all the cells and was left for 30 min. After aspirating the fixative, crystal violet staining solution was directly added for staining for 30 min. Finally, the residual stain was washed off with PBS twice, and the plate was air-dried for photography and colony counting.

### In vivo tumor growth assay

After one week of acclimatization in an SPF environment, male C57BL/6 or BALB/c nude mice aged 4–6 weeks were randomly divided into two groups: the normal saline control group and the oncolytic virus treatment group (10⁷ plaque-forming units [PFU] per mouse, which was selected based on established preclinical studies and aligned with doses used in clinical trials of OHSV2 [15, 38]), with five mice in each group. Bladder cancer cells were digested with 0.25% EDTA trypsin and adjusted to the following concentrations: MB49 at 5 × 10⁶ cells per 100 µL and T24 at 1 × 10⁷ cells per 100 µL (for BALB/c nude mice, a 1:1 mixture of PBS and Matrigel [ABW, China] was used). The cell suspensions were kept on ice to maintain a single-cell state.

For inoculation, the axillary skin of the mice was disinfected, and the cell suspension was slowly injected subcutaneously using an insulin syringe. When the tumor volume exceeded 100 mm^3^ (day 0), treatment was initiated with intratumoral multi-point injections of 100 µL of the corresponding drug. The treatment was repeated on days 3 and 6, for a total of three doses. The TLR4 agonist RS09 (MCE, USA) was administered intraperitoneally at a dose of 2 mg/kg per injection every day from day 0. During the experiment, tumor volume calculated using the formula:

$$Volum\text{ } = Lengt\text{h }diameter \, \times {Widt\text{h }diamter}^{2} \, \div {2}$$

Mouse body weight was regularly monitored. On day 9, when the experiment was terminated, the tumor tissues were collected for measurement of mass and subsequent analysis.

### Tumor dissociation and flow cytometry

Excised tumor tissues were immediately placed on ice. A dissociation solution was prepared by supplementing serum-free DMEM with 0.1 mg/mL deoxyribonuclease I (bovine pancreas) (MCE, USA), 1 mg/mL collagenase (Sigma-Aldrich, USA), and 0.1 mg/mL hyaluronidase (Sigma-Aldrich, USA). Tissues were finely minced and digested in 2 mL of this solution for 1 h at 37 °C. The digested mixture was gently homogenized, passed through a 200-mesh sieve, and centrifuged at 400 × g for 5 min at 4 °C to pellet cells. After red blood cell lysis, a single-cell suspension was obtained for subsequent staining.

Cell surface/intracellular markers were stained according to standard protocols. Prior to acquisition, cells were filtered through a 400-mesh sieve. Samples were analyzed on a CytoFLEX flow cytometer (Beckman Coulter, USA), and data were processed using FlowJo software (v10.8.1). Detailed information on antibodies and reagents is provided in sTable 1.

### Hematoxylin and eosin (H&E) staining and histopathological evaluation

Paraffin-embedded tissue Sections (5 μm) from major organs (heart, liver, spleen, lung, and kidney) were deparaffinized in xylene and rehydrated through a graded ethanol series. Subsequently, the sections were stained with hematoxylin solution for 5–8 min, differentiated briefly in 1% acid ethanol, and blued in running tap water. After counterstaining with eosin (Solarbio, China) solution for 1–3 min, the sections were dehydrated, cleared, and mounted with neutral balsam.

For histopathological assessment, the H&E-stained slides were examined in a blinded manner by an experienced researcher. The evaluation focused on the preservation of normal tissue architecture, absence of necrosis or significant apoptosis, and lack of pronounced inflammatory infiltrates, hemorrhage, edema, or other pathological alterations. The criterion for “no obvious abnormality” was defined as the absence of the aforementioned pathological changes when compared to the tissues from the saline-treated control group.

### Apoptosis levels measurement

Cells were seeded at a density of 500,000 cells per well in a 6-well plate and cultured for 24 h after intervention treatment.

Apoptosis staining: The supernatant and PBS washes were collected, and the cells were digested with trypsin and centrifuged to obtain cell pellets. Each group was resuspended in Binding Buffer. For single staining, 5 µL of Annexin V-APC or PI dye (KeyGEN, China) was added, while for double staining, both dyes were added simultaneously. The cells were stained at room temperature in the dark for 10 min.

After staining, the cells were filtered through a 200-mesh filter into a flow cytometry tube and were analyzed on the flow cytometer.

### Immunohistochemistry staining

The paraffin sections were dewaxed with an environmentally friendly dewaxing agent in a gradient manner, followed by high-temperature antigen retrieval using EDTA buffer (pH 9.0) in a pressure cooker. After cooling, the sections were treated with 3% H₂O₂ to quench endogenous peroxidase activity and then incubated with 10% goat serum at 37 °C for 10 min to block non-specific binding sites. After incubation with the secondary antibody, the sections were stained with freshly prepared 3,3'-diaminobenzidine (DAB) chromogenic solution (Solarbio, China) and counterstained with hematoxylin (Solarbio, China) to visualize the nuclei. The results were observed under an optical microscope.

The information on antibody usage and dilution ratios is provided in sTable 2.

### RT-qPCR

Total RNA was isolated using the AFTSpin Tissue/Cell RNA Extraction Kit (ABclonal, China). The purity and concentration of RNA were measured using the NanoDrop One/OneC Spectrophotometer (Thermo Fisher). cDNA synthesis was performed using the Hifair® V One-Step RT-qPCR Mix (Yeasen, China). The qPCR reaction system included Hieff® SYBR Green Premix (Yeasen, China) and was run on the QuantStudioTM 6 Flex Real-Time PCR System (Applied Biosystems). The reaction program was as follows: initial denaturation at 95 °C for 5 min; 40 cycles of amplification at 95 °C for 10 s, 55°C–60 °C for 20 s, and 72 °C for 20 s; and a final dissociation curve acquisition. Data were normalized to the internal reference gene glyceraldehyde-3-phosphate dehydrogenase (*GAPDH*).

### Western blotting

Cell or tissue samples were lysed in RIPA buffer containing protease inhibitors (Solarbio, China) and phosphatase inhibitors (MedChemExpress, USA) to extract total protein. Protein concentration was determined using the bicinchoninic acid (BCA) method (Boster, China). Thirty micrograms of protein were loaded onto a 10% SDS-PAGE gel (Epizyme, China) and then transferred to a polyvinylidene difluoride (PVDF) membrane (Millipore, USA). The membrane was blocked at room temperature for 20 min using Boster blocking solution, incubated with primary antibodies overnight at 4 °C, and then with horseradish peroxidase (HRP)-conjugated secondary antibodies (1:10,000, ABclonal, China) for 1 h at room temperature. Finally, the proteins were visualized using a chemiluminescent detection system (Bio-Rad, USA).

The information on antibody usage and dilution ratios is provided in sTable 2.

### Multiplex cytokine assay

Concentrations of each factor were calculated using standard curves. Serum levels of multiple cytokines were measured using a multiplex bead-based immunoassay (ABplex, ABclonal, China) according to the manufacturer's instructions. Briefly, capture antibodies specific for each target cytokine were pre-coated onto distinct fluorescently encoded magnetic beads. The beads were mixed and incubated with 50 μL of serum samples, standards, or controls in a 96-well plate at room temperature for 2 h with gentle shaking. After washing with wash buffer, biotinylated detection antibodies were added and incubated for 1 h. Subsequently, streptavidin‑phycoerythrin (SA-PE) was added and incubated for 30 min. The plate was then washed, the beads were resuspended, and fluorescence intensity was measured using a flow‑based multiplex detection system.

### Bioinformatic analysis of public transcriptomic datasets

To contextualize the expression patterns observed in our study, we analyzed publicly available transcriptomic datasets from other cancer models treated with HSV-based oncolytic viruses. Dataset GSE298912 (prostate cancer, OHSV2-treated) [39], GSE186548 (colorectal cancer, HSV-2-based OV-treated) [40], and GSE262913 (oral cancer, HSV-2-based OV-treated) [41] were downloaded from the Gene Expression Omnibus (GEO) database. For each dataset, we extracted the normalized expression values for genes of interest (TLR4, NLRP3, CASP1, GSDMD) from the control and treatment groups. Data visualization and result summarization were conducted using R (v4.3.0).

### Statistical analysis

Flow cytometry data were analyzed using FlowJo software (v10.8.1, BD Life Sciences) for cell population quantification and quality control. Graphical representations and statistical analyses were generated with GraphPad Prism (v9.0, GraphPad Software). Between-group comparisons were performed using two-tailed Student's t-tests, with statistical significance set at *p* < 0.05. Data are presented as mean ± SD. *p < 0.05, **p < 0.01, ***p < 0.001, ****p < 0.0001.

## Supplementary Information

Supplementary Material 1.

## Data Availability

Qi-Dong Xia (qidongxia_md@163.com) is the lead contact for further information and resource requests. Data reported in the paper and additional information for data reanalysis can be shared by the lead contact upon reasonable request.
