# Supplementary material for: Oncolytic virus OHSV2 induces pyroptosis in bladder cancer cells via the TLR4/NLRP3/Caspase-1/GSDMD pathway
Source: Mol Biomed. 2026 Jul 28;7:119. doi: 10.1186/s43556-026-00506-4 (PMC13415730; doi:10.1186/s43556-026-00506-4)
Supplement: Supplementary file 1 — Supplementary Material 1. [file 43556_2026_506_MOESM1_ESM.pdf]

Supplymentary information

Title: Oncolytic virus OHSV2 induces pyroptosis in bladder cancer cells via the TLR4/NLRP3/Caspase-1/GSDMD pathway

Author list: Jinzhou Xu[1, 2†], Yifan Xiong[1, 2†], Chenqian Liu[1, 2, 3†], Jianxuan Sun[1, 2], Ye An[1, 2], Zhiyu Xia[1, 2], Binlei Liu[4*], Jia Hu[1, 2*], Qidong Xia[1, 2*], Shaogang Wang[1, 2*].

Affiliation：

1. Department and Institute of Urology, Tongji Hospital, Tongji Medical College, Huazhong University of Science and Technology, Wuhan, China.
2. Hubei Provincial Clinical Research Center for Minimally Invasive Treatment of Urology, Wuhan, China
3. Laboratory of Signaling and Gene Regulation, Cecil H. and Ida Green Center for Reproductive Biology Sciences, University of Texas Southwestern Medical Center, Dallas, USA
4. National "111" Center for Cellular Regulation and Molecular Pharmaceutics, Key Laboratory of Fermentation Engineering (Ministry of Education), Hubei Provincial Cooperative Innovation Center of Industrial Fermentation, Hubei Key Laboratory of Industrial Microbiology, Hubei University of Technology, Wuhan, China.
5. **footnotes:**

[†]**Co-first authorship:** These authors contributed equally to this work

***Corresponding author:** Shaogang Wang (sgwangtjm@163.com), Qidong Xia (qidongxia_md@163.com), Jia Hu (jiahutjm@163.com), Binlei Liu (liubinlei@binhui-bio.com)

Content

sTable 1. **Resource table for flow cytometry**

sTable 2. **Resource table for Antibodies**

Fig. S1. **In vitro detection of anti-tumor effect of OHSV2 in bladder cancer.**

Fig. S2. **Validation of the oncolytic virus OHSV2 in vivo.**

Fig. S3. **In vivo safety evaluation and flow cytometry gating strategy.**

Fig. S4. **OHSV2 intervention induces pyroptosis in bladder cancer cells.**

Fig. S5. **NLRP3 expression in subcutaneous xenograft tumor tissues from nude mice.**

Fig. S6. **TLR4 expression, knockdown validation, and NF-κB pathway involvement.**

Fig. S7. **Gating strategy, additional cytokine analysis, and safety evaluation for combination therapy.**

Fig. S8. **Expression levels of TLR4, NLRP3, CASP1, and GSDMD in three independent datasets.**

Fig. S9. **Immunohistochemical analysis of the TLR4/NLRP3/Caspase-1/GSDMD pathway in combination therapy.**

Fig. S10. **Combination of OHSV2 with BCG enhances pyroptosis in a subcutaneous xenograft tumor model.**

sTable 1. Resource table for flow cytometry

Antibody	Clone	Supplier	Catalog No.
PerCP/Cyanine5.5 anti-mouse CD223 (LAG-3)	**C9B7W**	**BioLegend**	**125211**
APC anti-mouse CD366 (Tim-3)	**RMT3-23**	**BioLegend**	**119705**
Brilliant Violet 605™ anti-mouse CD279 (PD-1)	**29F.1A12**	**BioLegend**	**135220**
APC anti-human/mouse Granzyme B Recombinant	**QA18A28**	**BioLegend**	**372203**
Brilliant Violet 421™ anti-mouse TNF-α	**MP6-XT22**	**BioLegend**	**506328**
Brilliant Violet 650™ anti-mouse IFN-γ	**XMG1.2**	**BioLegend**	**505832**
PE/Dazzle™ 594 anti-mouse F4/80	**BM8**	**BioLegend**	**123146**
Alexa Fluor® 700 anti-mouse CD86	**PO3**	**BioLegend**	**105122**
PE anti-mouse CD11c	**N418**	**BioLegend**	**117308**
PE/Dazzle™ 594 anti-mouse IL-12/IL-23 p40 Antibody	**C15.6**	**BioLegend**	**505219**
PE anti-mouse Perforin	**S16009A**	**BioLegend**	**154306**
PerCP anti-mouse CD4	**GK1.5**	**BioLegend**	**100431**
Brilliant Violet 605™ anti-mouse I-A/I-E	**M5/114.15.2**	**BioLegend**	**107639**
FITC Rat Anti-Mouse CD8a	**53-6.7**	**BD Biosciences**	**553030**
PE-CF594 Hamster Anti-Mouse CD3e	**145-2C11**	**BD Biosciences**	**562286**
APC-Cy7 Rat Anti-Mouse CD45	**30-F11**	**BD Biosciences**	**561283**
Alexa Fluor 700 Hamster Anti-Mouse CD69	**H1.2F3**	**BD Biosciences**	**561238**
PerCP-Cy5.5 Hamster Anti-Mouse CD80	**16-10A1**	**BD Biosciences**	**560526**
PE-Cy7 Rat Anti-CD11b	**M1/70**	**BD Biosciences**	**552850**
BV785 anti-mouse NK-1.1	**PK136**	**Biolegend**	**108749**
FITC anti-mouse Ly-6G	**1A8**	**Biolegend**	**127606**
Brilliant Violet 421™ anti-mouse CD8a	**53-6.7**	**Biolegend**	**100753**
Other		Supplier	Catalog No.
Zombie Aqua™ Fixable Viability Kit		**BioLegend**	**423102**
Fixation/Permeablization Kit		**BD Biosciences**	**554714**
Purified Rat Anti-Mouse CD16/CD32		**BD Biosciences**	**553141**

sTable 2. Resource table for Antibodies

Immunohistochemistry staining

Antibody	Dilution	Supplier	Catalog No.
TLR4 Polyclonal antibody	1:200	Proteintech	19811-1-AP
Anti-NLRP3 Antibody	1:200	Boster	BA3677
Anti-Caspase-1 Rabbit pAb	1:400	Servicebio	GB11383
Gasdermin D (N terminal) Rabbit Polyclonal Antibody	1:200	HUABIO	ER1901-37
Goat Anti-Rabbit IgG H&L (HRP)	1:500	SeraCare	5220-0336

Western Blotting

β-Tubulin Rabbit mAb	1: 10000	Abclonal	A12289
GSDMD (Full length+N terminal) Rabbit pAb	1: 400	Abclonal	A10164
GSDME (Full Length+N terminal) Rabbit mAb	1: 3000	Abclonal	A28230
caspase-1 (14F468)	1: 200	Santa Cruz	sc-56036
Vinculin Rabbit mAb	1: 50000	Abclonal	A2752
TLR4 Polyclonal antibody	1: 1000	Proteintech	19811-1-AP
NLRP3 (D4D8T) Rabbit Monoclonal Antibody	1: 1000	Cell Signaling Technology	15101T

Fig. S1. In vitro detection of anti-tumor effect of OHSV2 in bladder cancer.

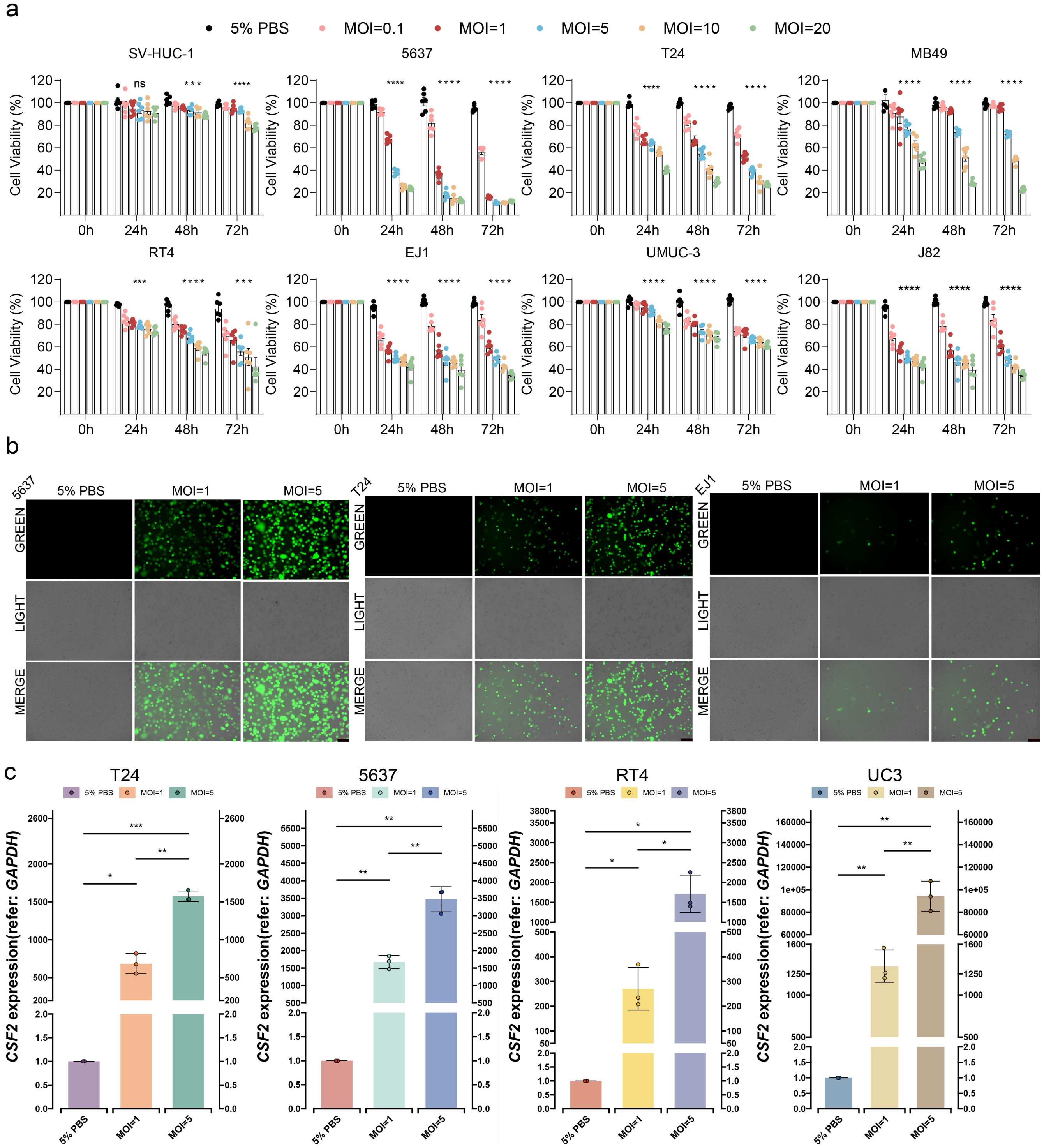

(a) Bar chart showing the viability of different bladder cancer cell lines treated with different concentrations of OHSV2 (n=6). (b) Detection of GFP expression in different bladder cancer cell lines after infection with GFP-OHSV2 in 5637, T24, and EJ1 (n=3). (c) Detection of *CSF2* gene (coding GM-CSF) expression levels in different bladder cancer cell lines after infection with OHSV2 (n=3). Data are presented as mean ± SD. Statistical significance was determined by unpaired two-tailed Student's t-test (*p < 0.05, **p < 0.01, ***p < 0.001, ***p < 0.0001).

Fig. S2. Validation of the oncolytic virus OHSV2 in vivo.

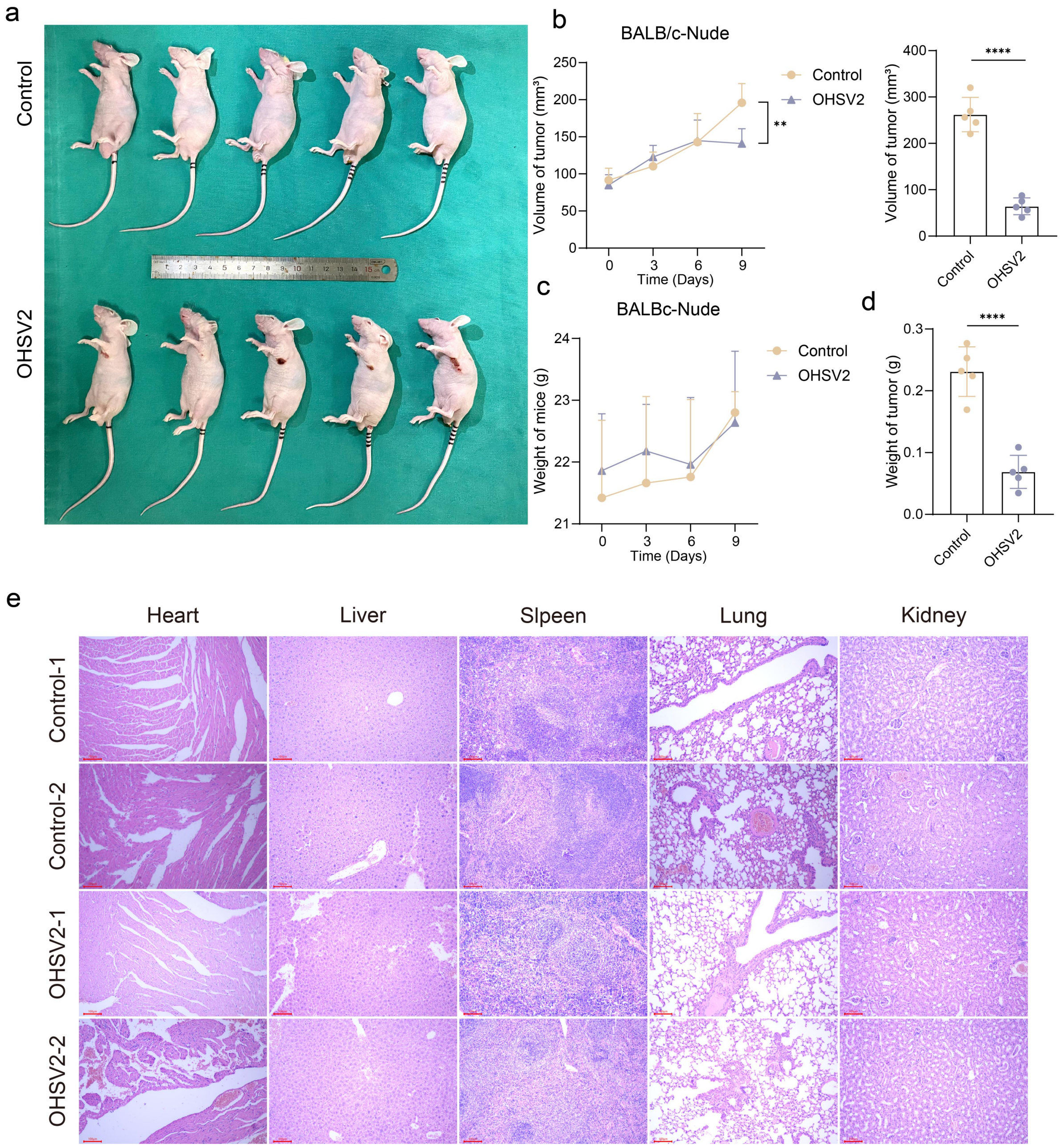

(a)In vivo images of tumors after BALB/c Nude mouse sacrifice (b) Line graph showing changes in tumor volume during the treatment period (n=6). (c) Line graph showing changes in mouse body weight during the treatment period for BALB/c Nude mice. (d) Bar graph comparing ex vivo tumor volumes and tumor weights after C57BL/6 mouse sacrifice (n=6) (e) Comparison of HE-stained structures of various organs (heart, liver, spleen, lung, kidney) after mouse sacrifice in BALB/c Nude. Data are presented as mean ± SD. Statistical significance was determined by unpaired two-tailed Student's t-test (*p < 0.05, **p < 0.01, ***p < 0.001, ***p < 0.0001).

Fig. S3. In vivo safety evaluation and flow cytometry gating strategy.

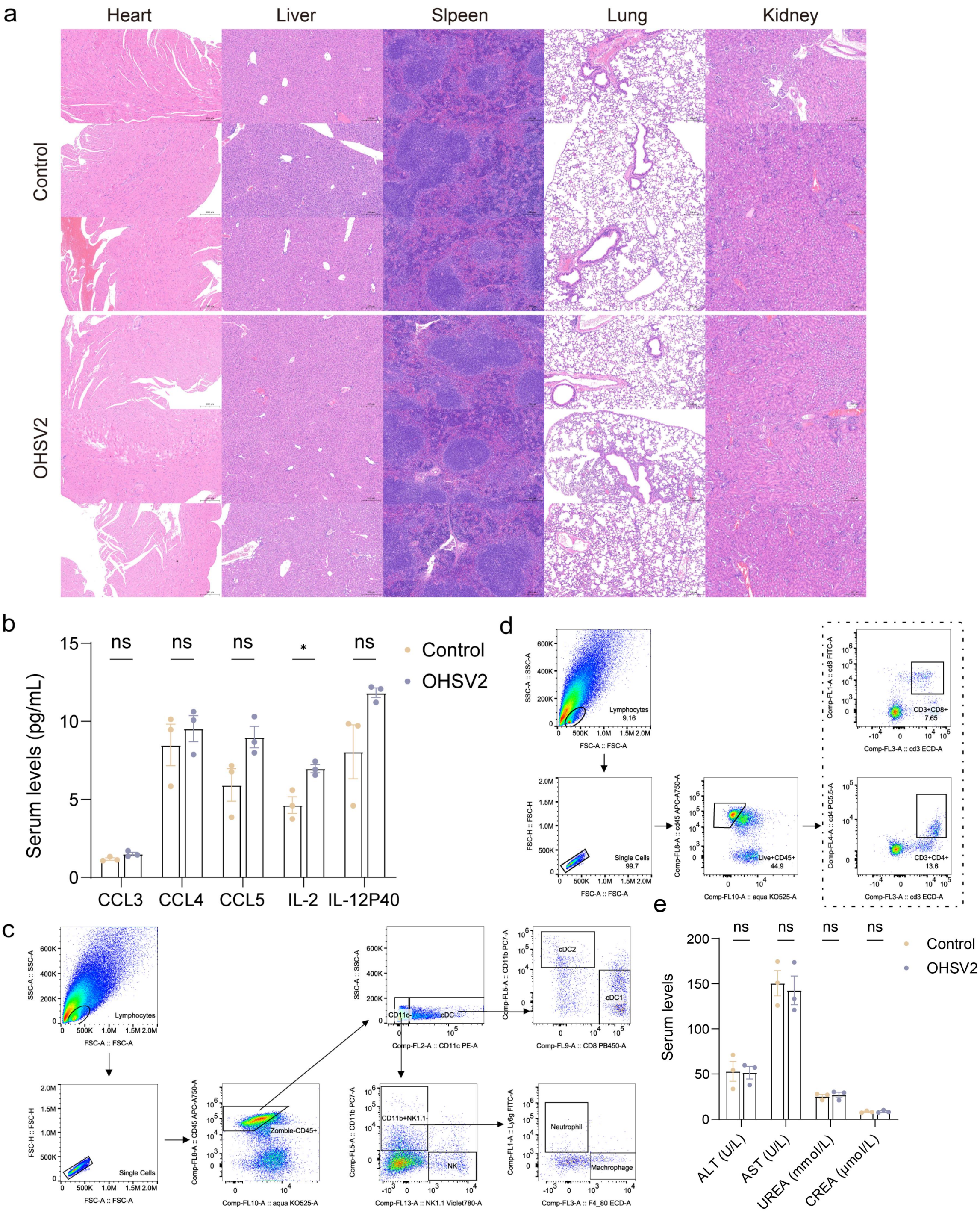

(a) Hematoxylin and eosin (HE) staining of heart, liver, spleen, lung, and kidney from C57BL/6 mice treated with PBS or OHSV2. Scale bars: 100 μm. (b) Multiplex cytokine analysis of mouse serum (additional data related to Fig. 2e) (n=5). (c, d) Representative flow cytometry gating strategies for immune cell subset analysis in tumor tissues. (e) Serum liver and kidney function tests showing no significant changes after intratumoral OHSV2 injection (n=3). Data are presented as mean ± SD. Statistical significance was determined by unpaired two-tailed Student's t-test (*p < 0.05, **p < 0.01, ***p < 0.001, ***p < 0.0001).

Fig. S4. OHSV2 intervention induces pyroptosis in bladder cancer cells.

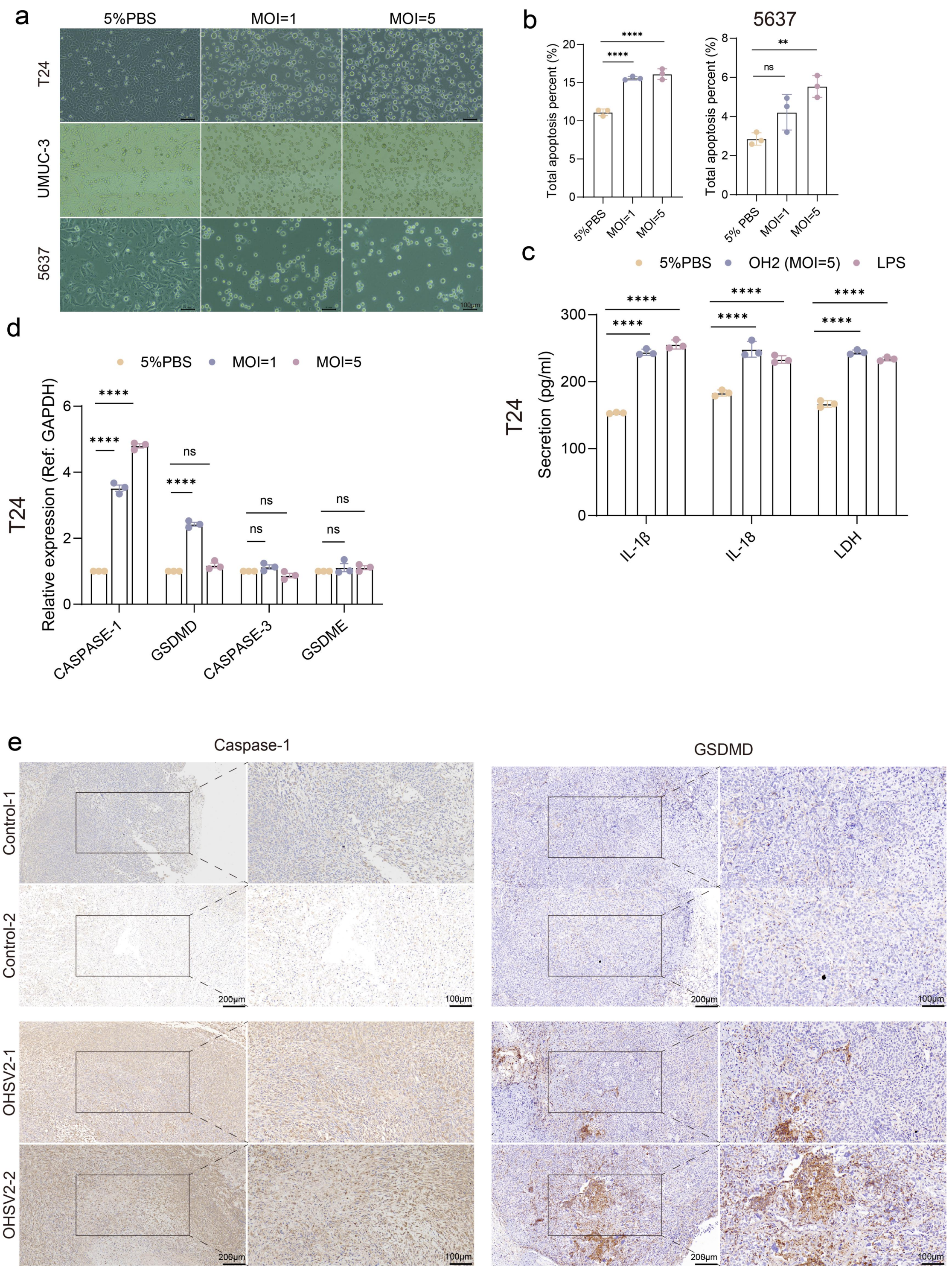

(a) Light microscopy images showing morphological changes of bladder cancer cells after OHSV2 treatment. (b) Statistical analysis of apoptosis rates detected by flow cytometry after OHSV2 treatment (n=3). (c) Expression levels of IL-18, IL-1β, and LDH in the cell supernatant

after OHSV2 treatment of T24 cells (n=3). (d) RT-qPCR analysis of the mRNA levels of *CASP1, GSDMD, CASP3, GSDME* in T24 cells treated with different concentrations (5% PBS, MOI=1, MOI=5) (n=3). (e) Immunohistochemistry analysis to detect the expression of Cleaved Caspase-1 and N-GSDMD proteins in subcutaneous xenograft tumor tissues from nude mice. Data are presented as mean ± SD. Statistical significance was determined by unpaired two-tailed Student's t-test (*p < 0.05, **p < 0.01, ***p < 0.001, ***p < 0.0001).

Fig. S5. NLRP3 expression in subcutaneous xenograft tumor tissues from nude mice.

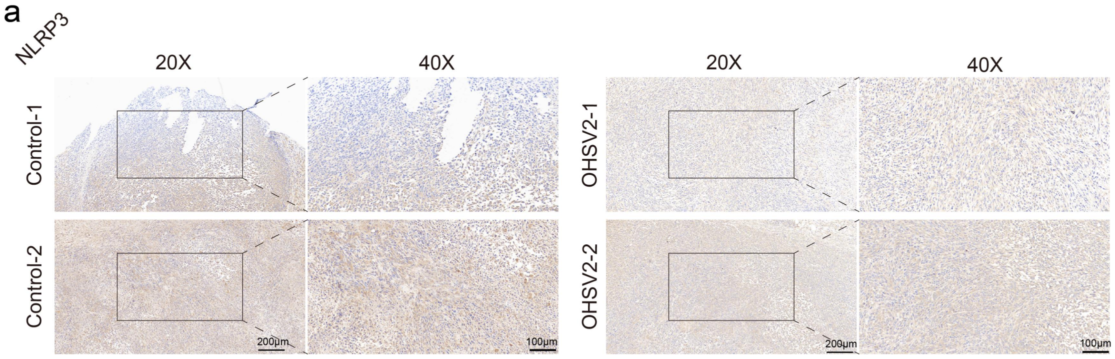

(a) Immunohistochemistry analysis to detect the expression of NLRP3 proteins in subcutaneous xenograft tumor tissues from nude mice.

Fig. S6. TLR4 expression, knockdown validation, and NF-κB pathway involvement.

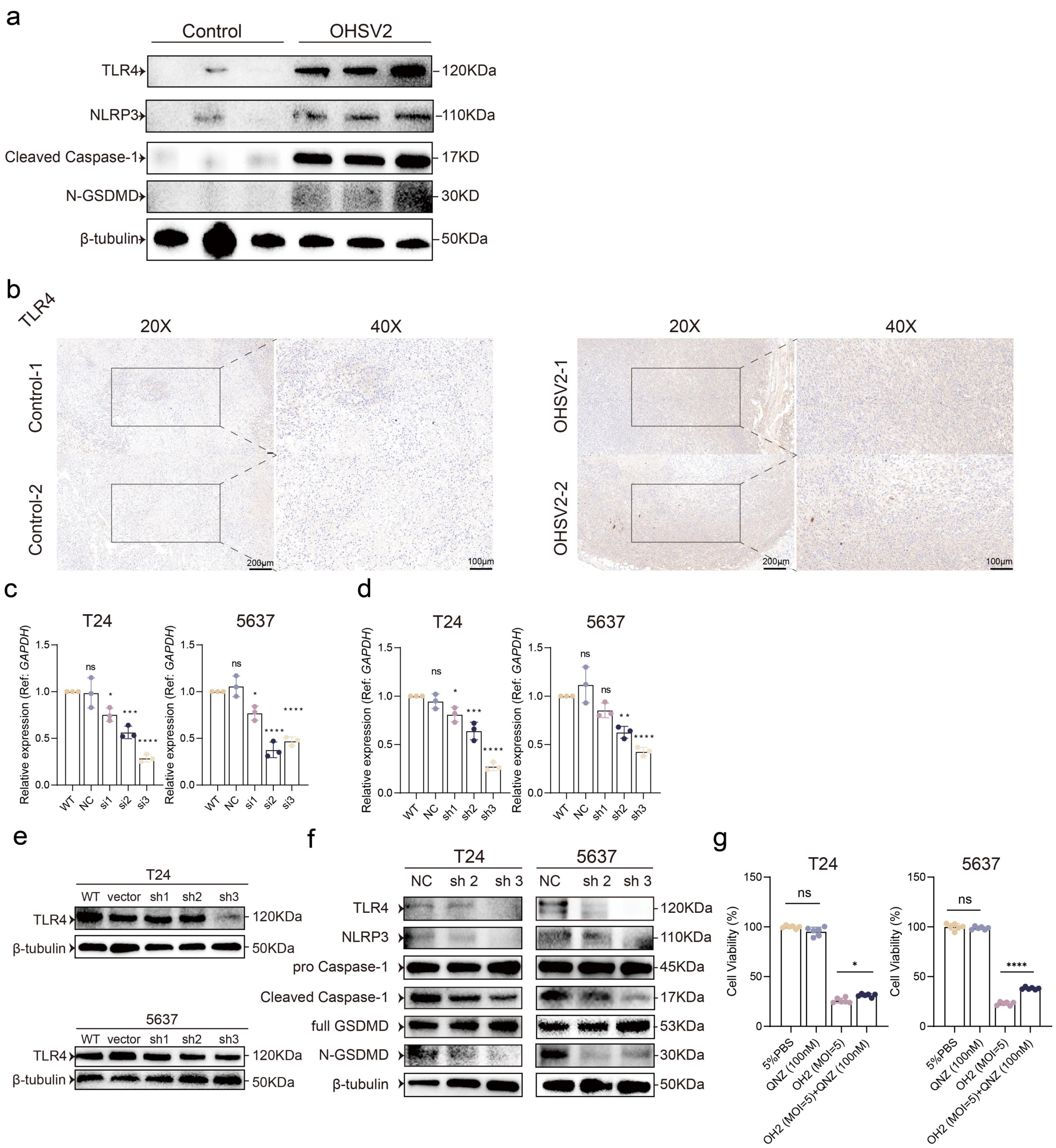

Western Blot (a) and immunohistochemistry (b) analysis to detect the expression of TLR4 proteins in subcutaneous xenograft tumor tissues from nude mice. (c, d) qPCR validation of *TLR4* knockdown efficiency using siRNA and shRNA (n=3). (e) Western blot validation of TLR4 knockdown efficiency using shRNA. (f) Western blot analysis of pyroptosis pathway after shRNA #2 and #3 knockdown. (g) CCK-8 assay showing cell viability after OHSV2 treatment combined with the NF-κB inhibitor QNZ (n=6). Data are presented as mean ± SD. Statistical significance was determined by unpaired two-tailed Student's t-test (*p < 0.05, **p < 0.01, ***p < 0.001, ***p < 0.0001).

Fig. S7. Gating strategy, additional cytokine analysis, and safety evaluation for combination therapy.

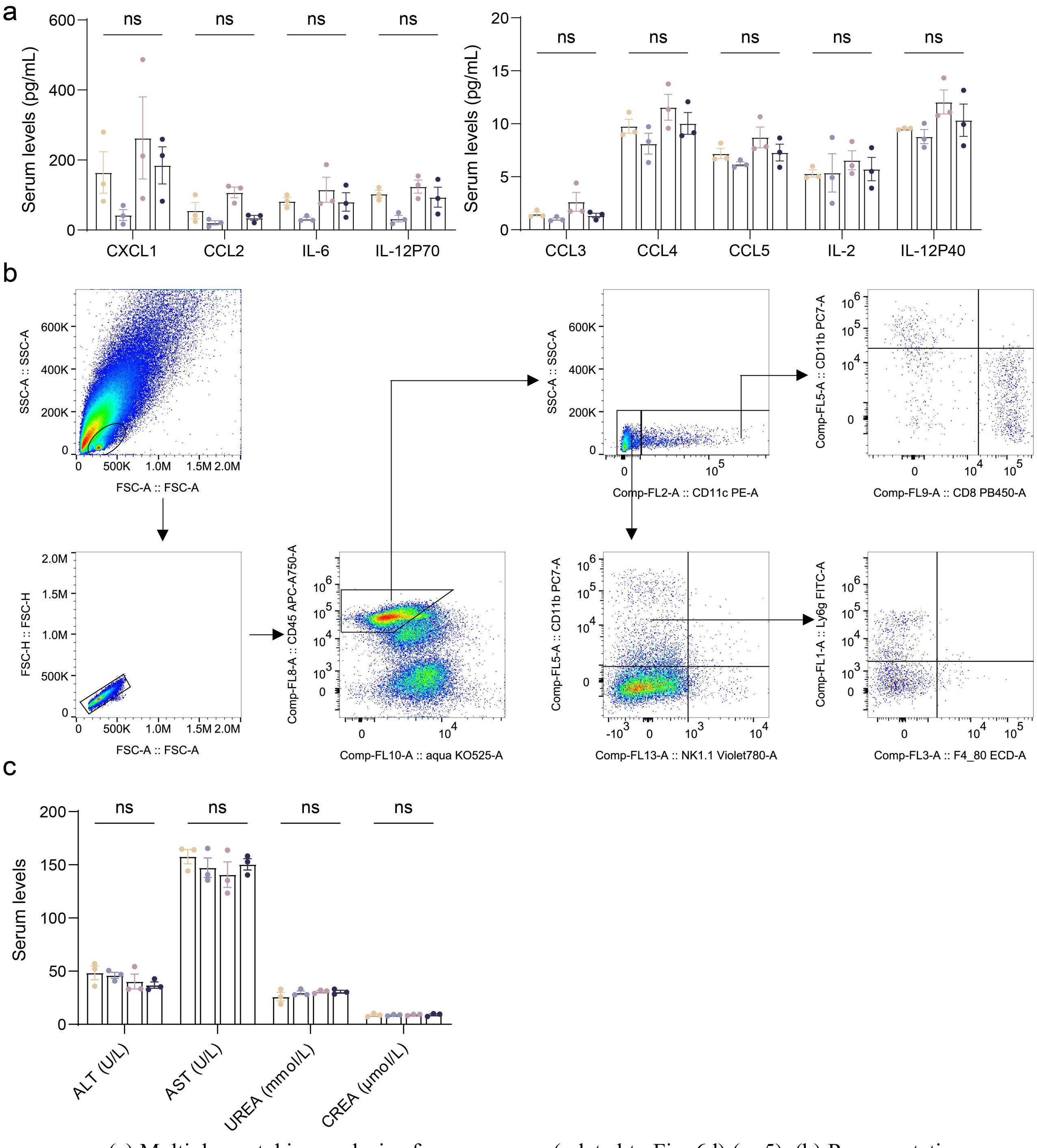

(a) Multiplex cytokine analysis of mouse serum (related to Fig. 6d) (n=5). (b) Representative flow cytometry gating strategy for immune cell subset analysis. (c) Serum liver and kidney function tests showing no significant changes after combination therapy (n=3). Data are presented as mean ± SD. Statistical significance was determined by unpaired two-tailed Student's t-test (*p < 0.05, **p < 0.01, ***p < 0.001, ***p < 0.0001).

Fig. S8. Expression levels of TLR4, NLRP3, CASP1, and GSDMD in three independent datasets.

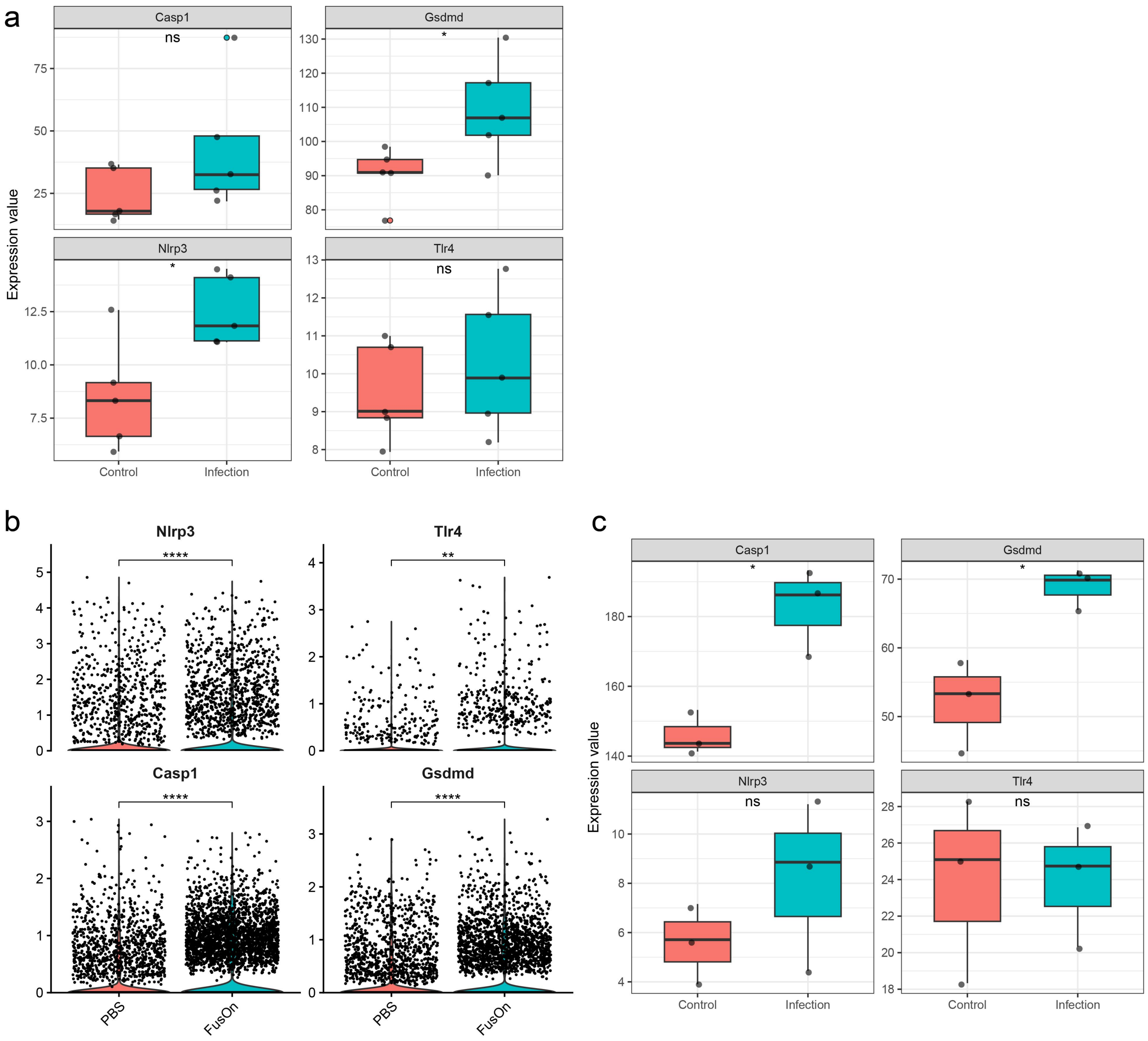

Expression levels of TLR4, NLRP3, CASP1, and GSDMD in three independent datasets: GSE298912 (a), GSE186548 (b), and GSE262913 (c). Data are presented as mean ± SD. Statistical significance was determined by unpaired two-tailed Student's t-test (*p < 0.05, **p < 0.01, ***p < 0.001, ***p < 0.0001).

Fig. S9. Immunohistochemical analysis of the TLR4/NLRP3/Caspase-1/GSDMD pathway in combination therapy.

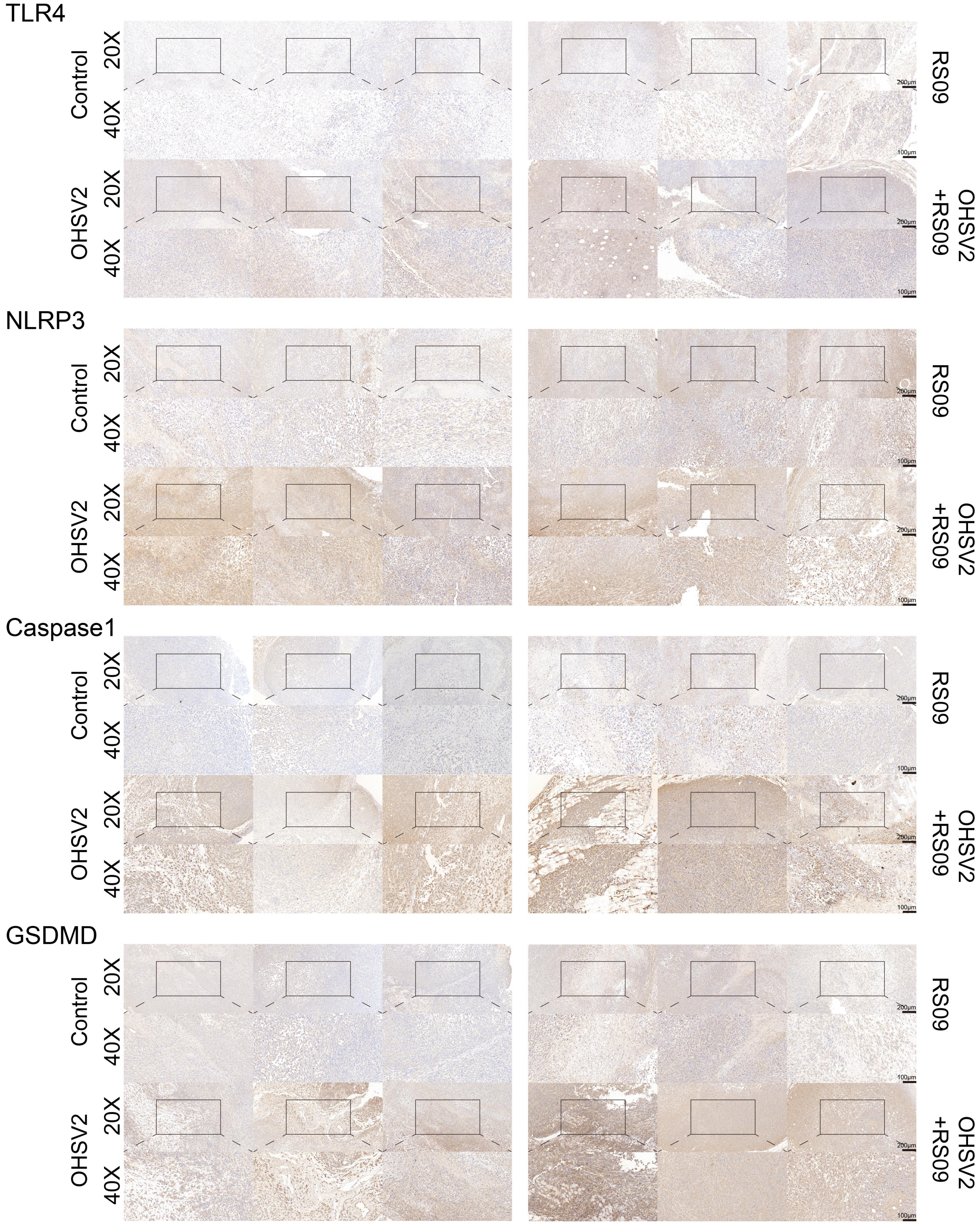

Fig. S10. Combination of OHSV2 with BCG enhances pyroptosis in a subcutaneous xenograft tumor model.

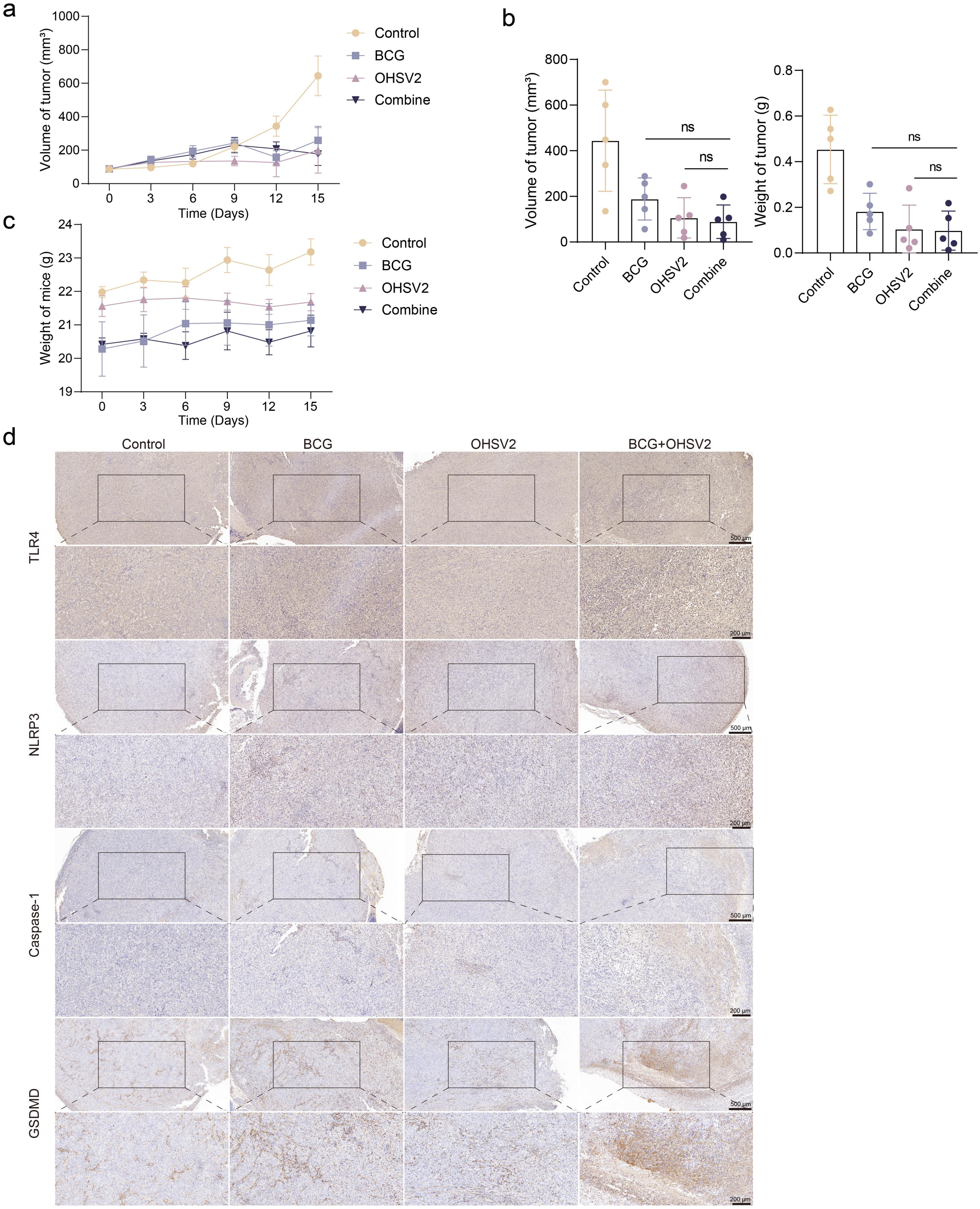

(a) Tumor growth curves of nude mice bearing T24 xenografts treated with PBS, OHSV2, BCG, or OHSV2 + BCG. (b) Comparison of ex vivo tumor volumes and tumor weights at the endpoint (n=5). (c) Body weight changes of mice during the treatment period (n=5). (d)

Immunohistochemical staining of TLR4, NLRP3, Cleaved Caspase-1, and N-GSDMD in tumor tissues. The combination group exhibited significantly enhanced activation of the pyroptosis pathway. Data are presented as mean ± SD. Statistical significance was determined by unpaired two-tailed Student's t-test (*$p < 0.05$, **$p < 0.01$, ***$p < 0.001$, ***$p < 0.0001$).
